# An Evolutionary Trace method defines functionally important bases and sites common to RNA families

**DOI:** 10.1371/journal.pcbi.1007583

**Published:** 2020-03-24

**Authors:** Ilya B. Novikov, Angela D. Wilkins, Olivier Lichtarge

**Affiliations:** 1 Department of Biochemistry and Molecular Biology, Baylor College of Medicine, Houston, Texas, United States of America; 2 Department of Molecular and Human Genetics, Baylor College of Medicine, Houston, Texas, United States of America; U Montreal, CANADA

## Abstract

Functional non-coding (fnc)RNAs are nucleotide sequences of varied lengths, structures, and mechanisms that ubiquitously influence gene expression and translation, genome stability and dynamics, and human health and disease. Here, to shed light on their functional determinants, we seek to exploit the evolutionary record of variation and divergence read from sequence comparisons. The approach follows the phylogenetic Evolutionary Trace (ET) paradigm, first developed and extensively validated on proteins. We assigned a relative rank of importance to every base in a study of 1070 functional RNAs, including the ribosome, and observed evolutionary patterns strikingly similar to those seen in proteins, namely, (1) the top-ranked bases clustered in secondary and tertiary structures. (2) In turn, these clusters mapped functional regions for catalysis, binding proteins and drugs, post-transcriptional modification, and deleterious mutations. (3) Moreover, the quantitative quality of these clusters correlated with the identification of functional regions. (4) As a result of this correlation, smoother structural distributions of evolutionary important nucleotides improved functional site predictions. Thus, in practice, phylogenetic analysis can broadly identify functional determinants in RNA sequences and functional sites in RNA structures, and reveal details on the basis of RNA molecular functions. As example of application, we report several previously undocumented and potentially functional ET nucleotide clusters in the ribosome. This work is broadly relevant to studies of structure-function in ribonucleic acids. Additionally, this generalization of ET shows that evolutionary constraints among sequence, structure, and function are similar in structured RNA and proteins. RNA ET is currently available as part of the ET command-line package, and will be available as a web-server.

## Author summary

Traditionally, RNA has been delegated to the role of an intermediate between DNA and proteins. However, we now recognize that RNAs are broadly functional beyond their role in translation, and that a number of diverse classes exist. Because functional, non-coding RNAs are prevalent in biology and impact human health, it is important to better understand their functional determinants. However, the classical solution to this problem, targeted mutagenesis, is time-consuming and scales poorly. We propose an alternative computational approach to this problem, the Evolutionary Trace method. Previously developed and validated for proteins, Evolutionary Trace examines evolutionary history of a molecule and predicts evolutionarily important residues in the sequence. We apply Evolutionary Trace to a set of diverse RNAs, and find that the evolutionarily important nucleotides cluster on the three-dimensional structure, and that these clusters closely overlap functional sites. We also find that the clustering property can be used to refine and improve predictions. These findings are in close agreement with our observations of Evolutionary Trace in proteins, and suggest that structured functional RNAs and proteins evolve under similar constraints. In practice, the approach is to be used by RNA researches seeking insight into their molecule of interest, and the Evolutionary Trace program, along with a working example, is available at https://github.com/LichtargeLab/RNA_ET_ms.

This is a *PLOS Computational Biology* Methods paper.

## Introduction

Functional non-coding (fnc)RNAs are a broad class of functional macromolecules that regulate transcription and translation, maintain genome stability [1], and play a role in diseases. They are found across evolution and include both classical as well as several new forms discovered over the past 30 years. The well-known classical RNAs primarily concern translation: they are ribosomal (r)RNA, transfer (t)RNA, small nucleolar (sno)RNA, and the tRNA maturation enzyme RNAse P. The novel RNA classes span self-splicing ribozymes that control viral replication, riboswitches that regulate small molecule metabolism in bacteria, small regulatory RNAs (microRNAs) that regulate mRNA translation in eukaryotes, and, most recently, long non-coding (lnc)RNAs that impact pre- and post-transcriptional gene expression [2]. Thus, functional non-coding RNAs are diverse and contribute significantly to cell metabolism. Critically, fncRNAs have been linked to human disease. For example, mutations in mitochondrial RNAse P are associated with cartilage-hair hypoplasia [3], deletion of promoter that drives expression of HBII-85 snoRNAs contributes to Prader-Willi syndrome [4], and mutations in hTR, RNA component of DNA telomerase, promote Dyskeratosis congenita [5]. Furthermore, small regulatory RNA are perturbed in cancer, in cardiovascular diseases, and neurodegenerative disorders [6], and studies have shown that fncRNA expression is significantly disturbed in cancer cell lines [7]. Long non-coding RNA MALAT1 has been directly linked to metastasis in lung and gastric cancer [8, 9]. These and other fncRNAs represent an entirely new class of druggable targets. Indeed, a number of inhibitors have already been developed to target pathogenic fncRNA, including riboswitches [10] and the ribosome [11]. Given the growing recognition of the role of fncRNA in human health [12], it is important to understand the determinants of function in these molecules.

To understand fncRNA structure and function and target them for therapy, a central question is which nucleotides in a given molecule contribute to function? Answers have thus far relied on structure determination and targeted mutagenesis. First, secondary or tertiary RNA structures are solved by any number of wet-lab techniques, such as x-ray crystallography, NMR, and enzymatic or chemical probing [13], or via *in silico* algorithms [14–18]. Based on the structure model, specific nucleotides may then be targeted for mutagenesis, as in [19]. This classical experimental paradigm is resource intensive and contingent on suitable biochemical assays, cell lines, and viable mutants.

In protein research, the similar challenge of identifying functionally important amino acid residues had been effectively addressed by the predictive computational methods, most notably Evolutionary Trace, which is the single most-validated approach [20]. However, in RNA research, there are currently no well-validated computational alternatives to the experimental paradigm (one exception being the protein-centric ConSurf web-tool that recently added the ability to score conservation of nucleic acid sequences [21]). Because the field is so nascent, most RNA sequence analysis tools, such as GERP++ and PhastCons [22, 23], are used primarily in genomic context to identify novel exons or ncRNAs, and in practice, they are not applicable to single-nucleotide functional analysis of individual RNA molecules.

Furthermore, the traditional purpose of sequence analysis in RNA has been to model secondary and tertiary structure via detection of canonical Watson-Crick base pairing. The first studies of homologue co-variation led to secondary structures of tRNA [24], 5S rRNA [25], and self-catalytic introns [26]. Structure prediction with aid of RNA sequence analysis further evolved with context-free grammar algorithms [27], and the recent advances in the field deal with prediction of non-canonical long-range tertiary contacts in larger molecules [28]. Unlike ET, these methods are primarily aimed at structure prediction, and do not directly provide analysis of evolutionary importance on single-nucleotide level.

**Fig 1:**
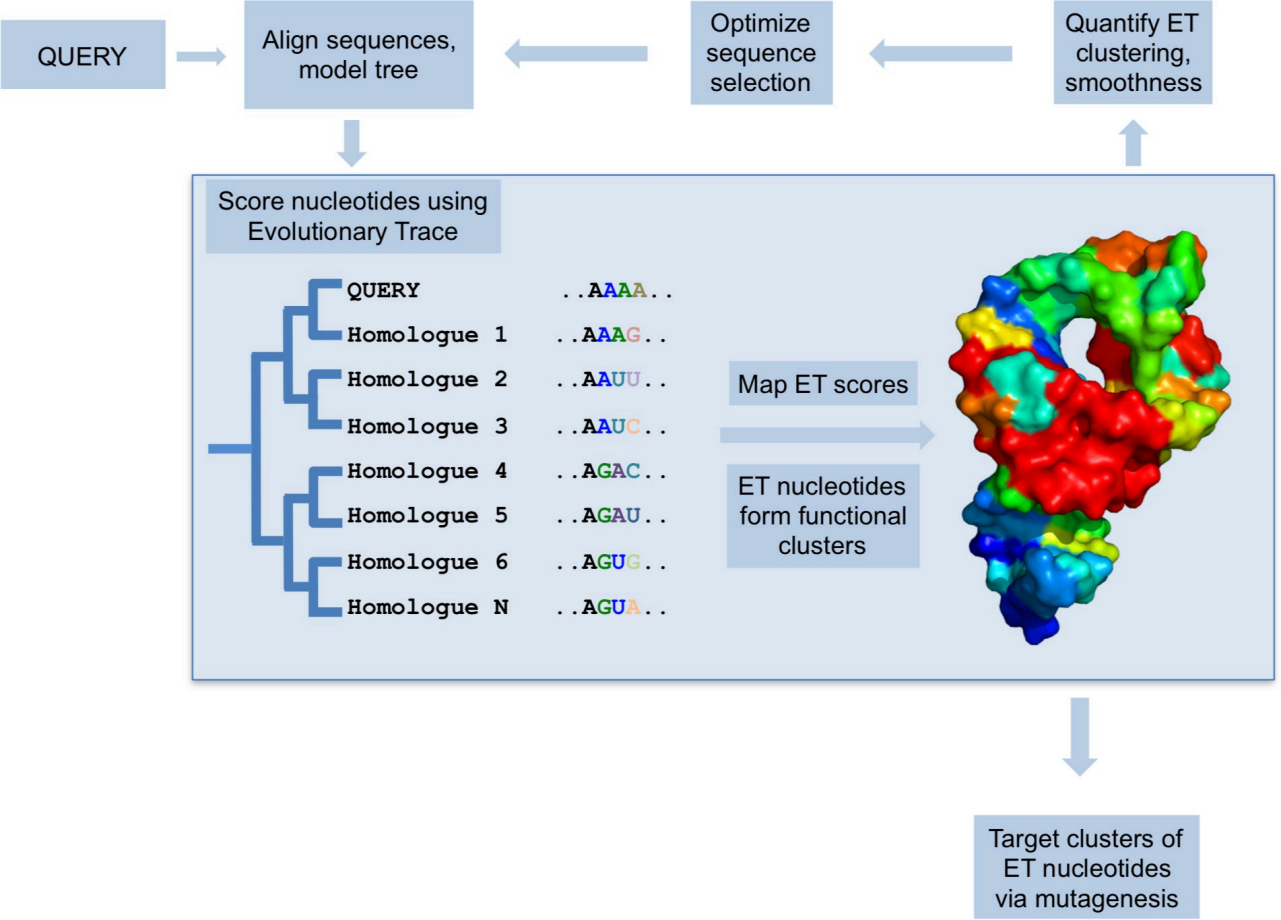
The Evolutionary Trace model. For a set of homologues, ET quantifies correlation between phylogenetic tree divergence and sequence variation. ET nucleotides, where the correlation is highest, are considered evolutionarily- and functionally-important. They cluster on the structure and predict functional sites. Furthermore, the quality of structural clustering by ET nucleotides can be measured, and then optimized to improve functional site prediction.

To address this need, we sought to adapt Evolutionary Trace [29, 30] to predict functional nucleotides in RNA from their evolutionary history. Evolutionary Trace is a method to identify functionally important residues in proteins. It correlates sequence variations with evolutionary divergences in order to rank sequence positions as more (or less) important to function (Fig 1). In so doing, ET makes two assumptions. First, that sequence variations during evolution and speciation are akin to sampling the sequence-function space via wet lab mutations. Second, that the depth of divergence between two sequences is commensurate with their functional difference, that is, this depth is a quantitative assay of functional distance. If so, a systematic tally of the variations, at any given position of a multiple sequence alignment, that track mostly with deep (or small) phylogenetic divergence, enable ET to assign a greater (or lesser) relative rank of evolutionary importance to each sequence position. More recently, it was recognized that such systematic coupling of *variations* in sequence space (genotype) with *variations* in evolution (fitness space) can be formally recast as a gradient of the evolutionary mapping of genotypes onto the fitness landscape [31]. Viewing ET as the gradient of the evolutionary landscape, presumably a foundational feature of biology, helps explain that the relatively simple *in silico* process of tracing phylogenetic trees and alignments of homologous sequences (see Methods) leads to varied and useful insights into the molecular basis of protein function. By targeting mutations to top-ranked sequence positions (so-called ET residues), ET-guided studies identify protein-protein interaction interfaces [32–34], allosteric [35] and ligand binding sites [36], recode ligand specificity [35], designed functionally-active peptides [37], and on a structural proteomic scale computationally predict the function of orphan proteins [38, 39].

A natural question is whether similar insights might be gathered for RNA by translating the ET formalism from amino acids sequences to nucleotide sequences. This is readily testable since in proteins top-ranked ET residues have well established general properties that underpin the method’s successes: (1) ET can rank amino acid sequence positions from most to least important, such that those in top 30^th^ percentile are called ET residues. (2) These ET residues cluster in the three-dimensional structure of the molecule [40] and (3) overlap its functional sites [32]. (4) Critically, the quality of the structural clustering of ET residues informs with quality of functional site overlap [41]. And finally, (5) the quality of overlap can be improved via optimized sequence selection that maximizes ET clustering [42] and minimizes rank differences of neighboring residues (a structural smoothing of ET ranks) [43].

**Fig 2:**
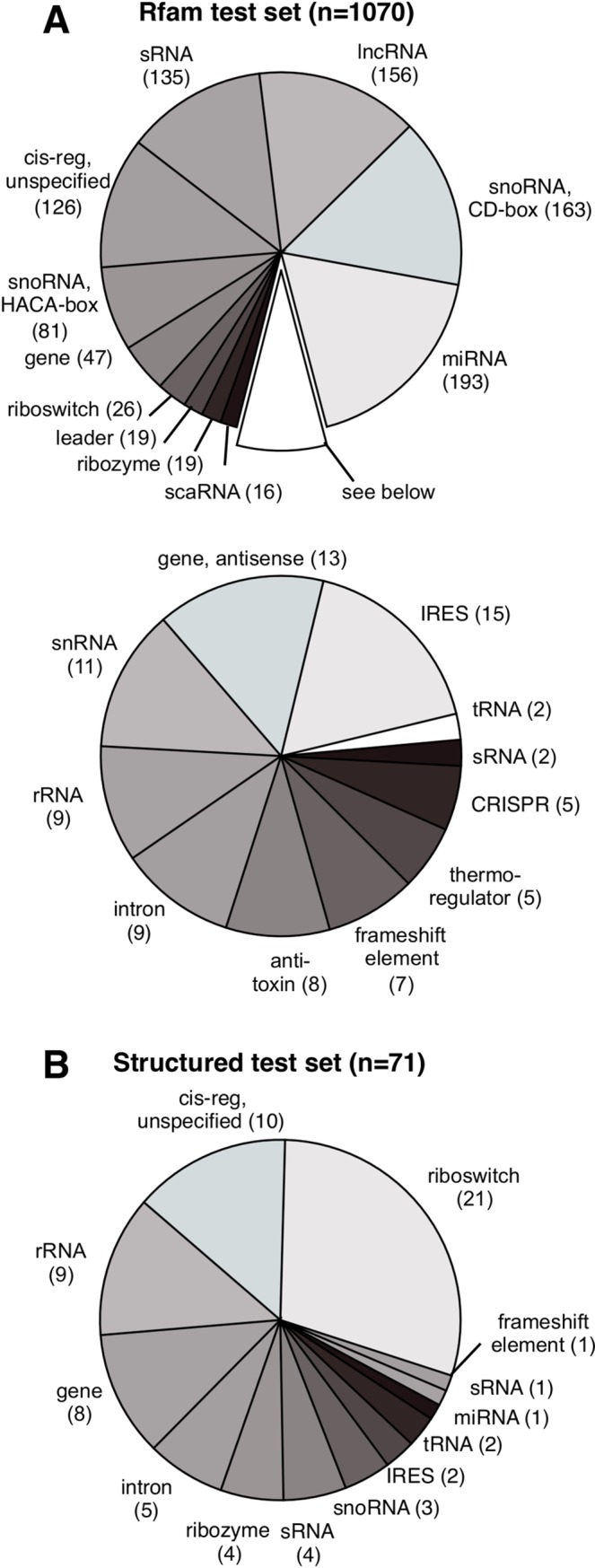
Rfam test set represents a broad selection of functional RNAs. Shown in (A) are Rfam families we used in our test set. In (B) is a subset of Rfam test families that map to high-resolution structures in the Protein Data Bank (PDB), allowing us to study three-dimensional clustering by ET nucleotides.

Therefore, to generalize the use of ET to RNA sequences, we sought to test whether ET nucleotides exhibit these five properties. We applied our tests to a representative set of RNA molecules from the Rfam database [44] (Fig 2A), and found that ET bases obey the same general rules as ET residues. In particular, we focused on a subset of well-characterized RNAs with known tertiary structures (Fig 2B), which account for 7% of our test set and are also fairly representative of overall RNA biology. In practice, the data show that Evolutionary Trace can be readily applied to multiple sequence alignments of homologous fncRNAs to identify nucleotides of functional importance.

## Methods

### Measuring nucleotide importance with real-value ET

To measure nucleotide importance of, we use Evolutionary Trace (Fig 1). The first step in the ET analysis is to construct a representative multiple sequence alignment (MSA) for the query sequence and its homologues. Here, we use the manually-curated seed alignments from the Rfam database [44], that each have at least 10 canonical sequences. The alignment is used to construct a UPGMA phylogenetic tree, and the two are then traced. ET iterates through the sequence columns and assigns a rank based on how closely the sequence variation within the column correlated with tree branching (note also that gaps are treated as a nucleotide). The first-generation ET algorithm [29, 32] expresses the rank as:

$$r_{i}=1+\sum_{n=1}^{N-1}\delta (n)\left\{ \begin{array}{c} \delta (n)=0\;if\;invariant\;within\;all\;groups\;g\;at\;level\;n\\ \delta (n)=1\;otherwise \end{array}\right.$$ (1)

where *r*_*i*_ is the rank of the residue at position *i*, *N* is the total number of sequences in the tree, and *N*−1 is the number of nodes. To compute *r*_*i*_, we iterate through every node *n* staring with one closest to the root (n = 1), and divide the sequences in the alignment into subgroups *g* based on the topology of the tree. Because the tree is binary, at node level *n*, the tree is divided into g = *n* groups. This division of tree into subgroups is shown in S1 Fig. We assign 0 to *δ(n)* if the residue at position *i* is invariant within all sequence groups g, and 1 otherwise. Thus, evolutionarily important nucleotides that are fixed within major branches will receive a lower absolute rank *r*_*i*_ than residues that continue to vary as the tree is traversed.

To illustrate the last point, consider two extreme examples. First, a sequence column that is entirely conserved will earn ET rank *r* = 1. Because the column is conserved, *δ*(*n*) is 0 at every step *n* in the summation function in Eq (1). Accordingly, the final sum is 0, and the column’s ET rank is 1, the lowest absolute ET rank (corresponding to the highest level of evolutionary importance). In contrast, consider a column that continues to vary until it is subdivided into N-1 subgroups (essentially, every sequence is in its own subgroup). For such a column, at every step *n*, *δ*(*n*) = 1, and the final rank *r* = *N*, the lowest possible rank for an alignment of *N* sequences.

This approach produces integer ranks, and suffers from treating each node as equally important, which is not always true. To address this, we developed real-value ET (rvET) [20, 30], an extension of the basic method, that uses information entropy to weight phylogenetic branches according to their sequence conservation:

$$r_{i}=1+\sum_{n=1}^{N-1}\frac{1}{n}\sum_{g=1}^{n}\left\{-\sum_{a=1}^{20}f_{ia}^{g}lnf_{ia}^{g}\right\}$$ (2)

where $f_{ia}^{g}$ is frequency of an amino (or nucleic) acid *a* found within the sequence group *g* that belongs to node *n*. Now, as we traverse the tree, we sum up and then average the information entropy for each of the sub-alignments *g* observed when we split the tree into *n* nodes. This allows us to produce better resolved ranks *r*_*i*_ that are more resistant to sequence inconsistencies, while still taking into account the phylogenetic history of the tree.

Of the two ET implementations described here, we use the more advanced real-value (rv)ET in his work. By applying rvET to an alignment of RNA homologues, we arrive at a relative ranking of evolutionary importance for every position in the alignment. In practice, we normalize ranks into percentile ranks, or coverage. The coverage of 5% includes the top 5% of the most highly ranked nucleotides, and so on. As a matter of convention, we refer to nucleotides ranked between 0% and 35% ET rank cutoff (35% ET coverage) as *ET nucleotides*. The choice of 35% is dictated by our prior experience in proteins, where protein residues ranked approximately between 20–30% ET rank coverage corresponded to the most critical functional sites of the protein.

### Measuring nucleotide clustering and overlap

To measure structural clustering by ET nucleotides, we developed the concept of Selection Clustering Weight (SCW), described in detail in [45]. Briefly, for a set of nucleotides *S*, Selection Clustering Weight, *w*, is the number of structural contacts formed by the members of *S*. To calculate *w*, we present the structure in form of adjacency matrix *A*:

$$A(i,j)=\left\{ \begin{array}{c} 1\;if\;d(i,j)\leq 4Å\\ 0\;if\;d(i,j)>4Å \end{array}\right.$$ (3)

where *d* is the distance between any two nucleotides *i* and *j*, and a contact is denoted by *A(i*, *j) = 1* if *d* is 4Å or less. Using selection function ***S****(x)* (which returns 0 if nucleotide *x* is not found in *S*, and 1 otherwise), we iterate over A and calculate *w*:

$$w=\sum_{i>j}^{L}S(i)S(j)A(i,j)$$ (4)

To assess the statistical significance of *w*, we compare it to the mean expected clustering weight, 〈*w*〉, by a random set of nucleotides of the same size as *S*. We express the difference between clustering weight of nucleotide set S, and random nucleotides, in form of a clustering z-score *z*_*c*_:

$$z_{c}=\frac{w-\langle w\rangle }{\sigma }$$ (5)

where *σ* is the standard deviation of 〈*w*〉. Both 〈*w*〉 and *σ* can be calculated analytically, as explained in [39]. Using this procedure, we calculated the statistical significance of clustering by ET nucleotides.

Similar to clustering z-score, we introduce overlap z-score *z*_*o*_ to assess how well ET nucleotides predict functionally-relevant sites. Given a pre-defined set of functional nucleotides of size *M* and a set of ET nucleotides of size *n* in a molecule of length *N*, we can use hypergeometric distribution to calculate mean expected overlap between the two:

$$m=n\frac{M}{N}$$ (6)

where *m* is the number of functional nucleotides one would expect in a selection of size n, if selection was random. The standard deviation of *m* is given by:

$$\sigma =\sqrt{\frac{nM(N-M)(N-n)}{N^{2}(N-n)}}$$ (7)

If the actual observed number of functional nucleotides in selected set is *k*, we can compute the z-score of overlap *z*_*o*_ as:

$$z_{o}=\frac{k-m}{\sigma }$$ (8)

Finally, in practice, we calculate both clustering and overlap z-scores over the entire range of ET ranks. We cumulatively bin nucleotides according to their ET rank so that selection ***S*** corresponds to all nucleotides at a certain ET rank threshold (ET coverage), and then measure the z-scores in each bin. As we are interested in top-ranked nucleotides, we average the z-scores in bins between 0 and 35% rank percentile (0 to 35% ET coverage), to get a single measure, $z_{c}^{35\%}$ or $z_{o}^{35\%}$. Note also that the maximum possible number of unique ranks and rank bins is *L*, the length of the query sequence. However, multiple nucleotides can share the same rank, which leaves a number of unique rank bins empty (not assigned to any nucleotides). We still incorporate these bins into the cumulative measure, by implicitly assigning to them the z-score from the closest valid bin.

### Measuring ET smoothness

In addition to quantifying ET clustering as clustering z-score, we also defined a global measure of clustering we refer to as *ET smoothness*, *SMT*. *SMT* reflects how smoothly ET ranks are distributed over the structure by tallying the rank difference of neighboring nucleotides:

$$SMT=\sum_{i,j}A(i,j){\left(x_{i}-x_{j}\right)}^{2}$$ (9)

where *A* is the adjacency matrix as described prior, and *x* is the ET rank of the nucleotides. In the original work addressing smoothness [43], we established that evolution tends to minimize difference in evolutionary importance between neighboring residues, because residues exert selective pressure on each other.

### Rfam test set

We traced seed alignments of 1070 families from the Rfam database, each family with a minimum of 10 unique canonical sequences (Fig 2A). Of these, 71 families with available high-resolution structures made up the structured test set that we tested for ET three-dimensional clustering (Fig 2B). Additionally, for a set of 15 families, we compiled a ‘golden standard’ to test for overlap with ET nucleotides. These Rfam test sets are listed in S1 Table. Additionally, the ribosomal ‘golden standard’ is listed on its own in S2 Table.

### Code availability

Evolutionary Trace code, compiled as a command-line utility, along with an example is available at https://github.com/LichtargeLab/RNA_ET_ms.

## Results and discussion

### Case study #1: Hammerhead ribozyme

**Fig 3:**
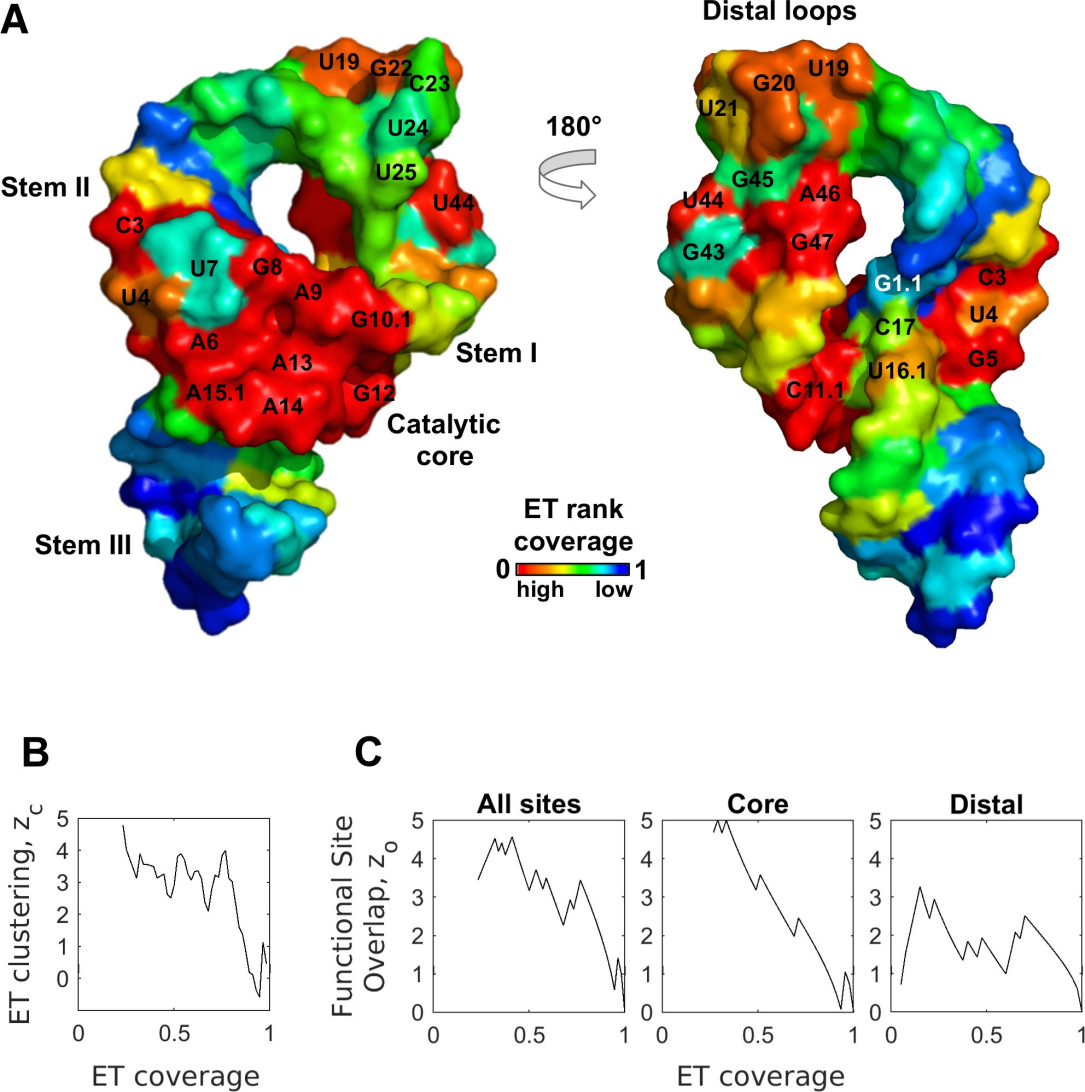
ET nucleotides cluster, and predict functional sites in the hammerhead ribozyme. (A) ET ranks mapped onto the structure of the hammerhead reveal clusters of ET nucleotides that overlap canonical functional sites (labeled nucleotides). (B) Clustering by ET nucleotides is statistically significant. (C) ET overlap z-score confirms that ET nucleotides inform both of the canonical functional sites. (To calculate site-specific $z_{o}^{35\%}$, we remove other known sites from consideration).

A first test of ET was the hammerhead ribozyme, a cis-cleaving structure most commonly found in plant viruses, which participates in rolling circle replication by cleaving the nascent transcript [46, 47]. The hammerhead motif is not confined to viruses, and new members of the family were recently described in bacteria and eukaryotes [46], where they may support tRNA and siRNA processing, ORF remodeling, and RNAi inhibition. The full-length hammerhead sequence is 60 nucleotides long, and the structure is defined by three short helices that meet at a junction (Fig 3A, PDBID 2QUS [48]). There are two main functional domains, the catalytic core that straddles the three-way junction and is responsible for cleavage, and a distal region defined by stem I-stem II tetraloops interactions, which promote efficient folding [49]. Nucleotides composing these two domains are labeled in Fig 3A. An evolutionary trace was computed on 26 non-redundant aligned sequences of class I and class II hammerhead ribozymes that represented the major branches of the plant viruses. This led to normalized ET rankings of each base position, from 0% (most evolutionary important) to 100% (least important), that were mapped on the structure in Fig 3A. This mapping highlights two clusters of ET bases defined by ET rank percentile below 35% (hereafter referred to as ET bases or ET nucleotides). The first of these clusters consists of 12 ET nucleotides and overlaps the catalytic junction. The second cluster consists of 5 ET nucleotides and overlaps the distal tetraloop region. The functional nucleotides and their respective domains are listed in Fig 3A.

Because clustering by ET residues is a defining feature of aminoacid ET, we assess the clustering of ET nucleotides in the hammerhead. Briefly, to quantify clustering of ET nucleotides, we calculate the number of pairwise structural contacts (distance of 4Å or less) between ET nucleotides, *w*, and compare it to the number of contacts formed by same number of nucleotides selected randomly, 〈*w*〉. Using the standard deviation associated with the random selection, σ, we then express the significance of clustering by ET nucleotides as a z-score $z_{c}=\frac{w-\langle w\rangle }{\sigma }.$ Quantitatively, the clustering z-score *z*_*c*_ is the number of standard deviations that separates the observed number of ET base contacts from the number of contacts expected randomly, and z-scores 2 and above denote statistical significance. See Methods for details.

Using this metric, we calculated clustering of hammerhead nucleotides, binned cumulatively according to their ET rank. We calculated *z*_*c*_ for every bin between 0 to 35% ET coverage (rank percentile), and found that ET nucleotides cluster with a mean z-score $z_{c}^{35\%}=3.9$ (Fig 3B). Not surprisingly, the clustering profile in Fig 3B is similar to the behavior of ET residues in proteins seen in our previous work [41]. We observed a high initial z-score, indicative of ET bases clustering to, hypothetically, form a major functional site, followed by a decline as we expand our ET coverage to include lower-ranked nucleotides. These data confirm that ET nucleotides cluster in the structure, a behavior we would not expect if nucleotide selection was random.

Next, we assessed the second major property of ET: the overlap between ET nucleotides and the molecule’s functional sites. To measure statistical significance of overlap in the hammerhead, we counted the number of active site bases in each ET bin, *k*, and compared this to the number of active site bases one expects to recover if selecting randomly, *m*. The random selection was modeled as a hypergeometric distribution, and had an associated standard deviation, which allowed us to convert *k* into a z-score of overlap *z*_*o*_ (see Methods for details).

We calculated overlap z-score for the hammerhead as a function of ET coverage, and it is shown in Fig 3C, with underlying data summarized in S3 Table. The first point on the curve (z_o_ = 3.6 at 23% ET coverage) corresponds to ET overlap with the catalytic core of the molecule. This is followed by two strong spikes, first corresponding to recovery of the most important nucleotides in the second functional site (z_o_ = 4.5 at ET coverage 32%), and then the recovery of remaining nucleotides in the catalytic core (z_o_ = 4.6 at 41% ET coverage). Thereafter, each additional spike corresponds to recovery of a functional nucleotide by a lower ET threshold. These data show that that ET nucleotides (0–35% range) strongly overlap hammerhead’s two active sites (mean z-score $z_{o}^{35\%}=3.6$). In addition to overlap z-score, we also measured quality of ET prediction in a more conventional manner using receiver-operator-characteristic (ROC) curves, and ET ranking recovers hammerhead active sites with AUC = 0.82 (S2A Fig).

Next, we examined the ET clusters in greater detail. The core 12-nucleotide ET cluster overlaps the 16-nucleotide catalytic site of the hammerhead [19] with a site-specific mean z-score $z_{o}^{35\%}=4.5$ and AUC of 0.87 (Fig 3B center, and S2B **Fig**). Notably, the ET cluster contains the key catalytic nucleotides G12 and G8 (both with ET rank percentile of 23%), which are the general base and acid in the cleavage reaction. The ET cluster is also enriched with thermodynamically costly unpaired nucleotides (A6, G5, U4), and nucleotides paired in the non-canonical Hoogsten fashion (C3 forms a Hoogsten pair with U7 and G8, C17 with A13 and U16.1). The highly ranked bases presumably fulfill a critical functional role maintained during evolution, leading to their top ET rankings. Critically, mutations in 11 of the 12 nucleotides in this ET cluster completely abolished cleavage activity [19]. These data confirm that the ET cluster directly overlaps the main functional site of the hammerhead.

Of note, four bases in the catalytic core (U16.1, C17, U7, and G1.1) are not part of the 12-nucleotide ET cluster, because their ET rankings of 36%, 52%, 77%, and 96% fall below the 35% threshold we used to define the ET cluster. While U16.1 straddles the 35% threshold and in practice would be considered part of the ET cluster, the three other bases bear closer examination. Their significantly lower ranks suggest that these positions may be under lesser functional pressure, or possibly that there is inherent functional resilience to mutations. To test this hypothesis, we examined carefully the catalytic mechanism of the hammerhead, and the functional role of these three nucleotides.

Although the two nucleotides G1.1 and C17 (ET rank percentile = 96% and 52%) directly participate in the reaction (C17 is the nucleophile and its activated 2’-hydroxy group attacks the phosphate group of sessile G1.1 [48]), the critical electron transfer path is along the sugar-phosphate backbone and not their nitrogenous base. As a result, neither the G1.1 nor the C17 nucleotide is under heavy selective pressure. Indeed, direct mutational studies showed that position 1.1 accommodate all four bases, and C17 could also accommodate guanine and adenine (20% reduction in activity [19]). Nevertheless, C17 forms a non-canonical Hoogsten pair with core nucleotide A13, and, perhaps as a result, cannot accommodate uracil (500-fold reduction in activity [19]). In keeping with this greater selective pressure, C17 has a substantially better ET rank (of 52%) than the sessile G1.1 (96%).

The other notable exception in the catalytic core is U7 (ET rank percentile = 77%). This base is nested among the 12 ET nucleotides, incongruent with its apparent mutational freedom. Yet, the exhaustive mutagenesis studies confirmed that U7 tolerates substitution [19]. While substituting any of the 12 ET nucleotides in the catalytic core reduces activity from 10- to 1000-fold, U7 mutations have no impact on the reaction rate. Thus U7 base identity is not structurally or functionally critical, in keeping with lower ET rank.

In contrast to these data, there is evidence that the last exception, U16.1 which straddles the ET threshold (ET rank = 36%), is critically functional. This base is positioned closely to core nucleotides G12 and C17 (general base and nucleophile in the cleavage reaction), and a recent study suggested that U16.1 could be responsible for coordinating Mg^2+^ in a binding pocket formed by the three bases [50]. The predicted role of the ion is to lower the p*K*_a_ of G12 to make it more reactive toward C17. Therefore, unlike the three low-ranked exceptions, U16.1 is probably functional, as reflected by its near-threshold rank. Together these data show that the ET ranks of the core catalytic nucleotides are remarkably consistent with the mutational and biochemical interpretation of their functional role.

Next, we examined the apical cluster, formed by the five ET nucleotides (U19, G20, G22, U44, and A46) in the stem I and II loops. This ET cluster overlaps hammerhead’s11-base tetraloop-tetraloop domain (labeled as **Distal region** in Fig 3A), which is important for the efficient folding of the hammerhead core [49]. Within the domain, ET nucleotides form the structurally critical links between the two stems: U19 of stem I forms a pair with A46 of Stem II, while G20 and G22 bond with G45. Notably, these interactions are the energetically unfavorable Watson-Crick/Hoogsteen pairs, suggesting that hammerhead maintains them through evolution because they are functional. The last ET nucleotide in this cluster, U44, does not form cross-stem interactions, suggesting it serves a different structural role. Of the remaining six (non-ET) bases in the domain, only two form non-canonical interactions, and one of them (U21) is ranked just under the ET threshold (ET rank percentile = 39%). These data show that ET discriminates between the more and less important nucleotides in this domain. Overall, ET bases overlap with the distal domain with a mean z-score $z_{o}^{35\%}=2.0$, and ET predicts this domain with AUC of 0.76 (Fig 3C right, and S2C Fig).

Notably, the two ET clusters are in accordance with the accepted two-step model for hammerhead folding. In the model, stem I and II of the distal region fold first, thereby promoting efficient folding of the catalytic core [51]. The catalytic core is universally conserved, and indeed ET performs very similar to sequence conservation when identifying it, as shown in S3A Fig (z-score of overlap) and S3B Fig (ROC curve).

The distal region, however, lacks obvious sequence conservation. As a result its discovery was delayed by several years because researchers focused exclusively on the conserved catalytic core. Ultimately, kinetic and chimeric studies in the full-length hammerhead [49, 51] revealed that tetraloops are a functionally important domain. Ranking bases according to sequence conservation fails to detect the tetraloop domain, and ET outperforms conservation both in the z-score measure (S3C Fig, mean overlap z-score $z_{o}^{35\%}=2.0$ vs 0.41), and the ROC AUC (S3D Fig, AUC = 0.76 vs AUC = 0.63). ET detects the distal tetraloops, because while they are fairly variable across the entire tree, they are conserved within their respective class I and class II branches. ET detects this pattern of base variation, resulting in greater predictive power. Conservation-based ranking, mapped on to the structure in S4 Fig, highlights this difference between ET and sequence conservation.

In summary, these data show that ET detects clusters of evolutionary-important bases that define functional domains of the hammerhead. The equatorial ET cluster overlaps the catalytic core of the hammerhead, and the apical ET cluster overlaps the most important bases in the tetraloop domain. Notably, ET outperforms conservation when scoring bases in the tetraloop domain, because it takes into account the evolutionary history of the molecule. Furthermore, where biochemical evidence is available for individual nucleotides, it is remarkably coherent with the nucleotide ranks assigned by ET.

### Case study #2: Bacterial ribosome

**Fig 4:**
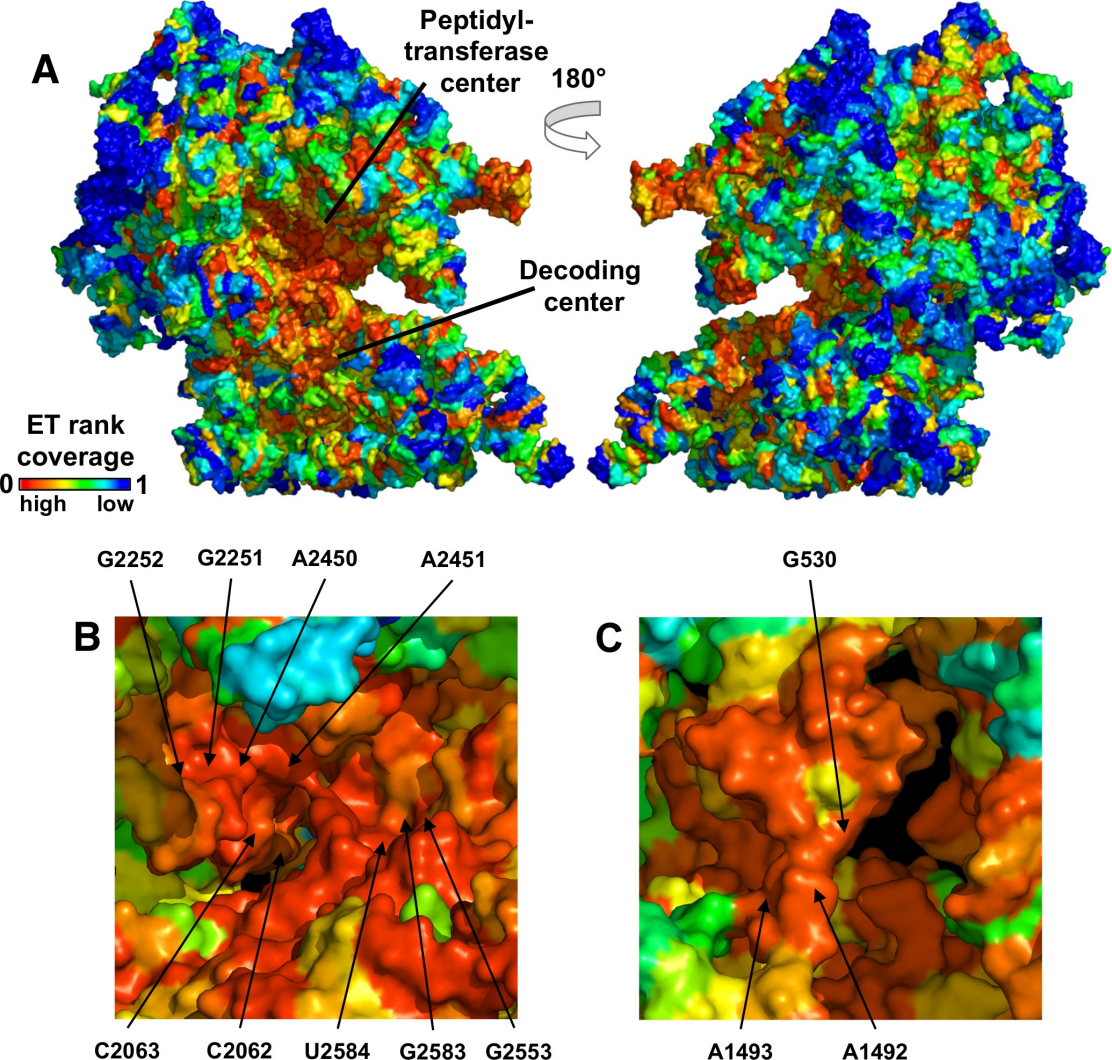
ET mapping reveals clusters that overlap active sites in the ribosome. ET ranks are mapped onto the structure in (A). Note the continuous ET cluster that spans both subunits and houses the peptidyl-transferase center (PTC) and the decoding center. Both are shown in detail in (B) and (C), where known catalytic nucleotides are labeled.

The second RNA model system to test ET was the bacterial ribosome. Ribosome is the universally-conserved ribonucleic complex that synthesizes polypeptides from mRNA templates. Mature bacterial ribosomes are comprised of two RNA molecules, the 16S and 23S ribosomal (r)RNA, as well as over 50 ribosomal proteins that bind the rRNA during assembly. The RNA component of the ribosome was originally thought to play a primarily structural role, but high-resolution crystal structures revealed that rRNAs are, in fact, responsible for the catalytic activity of the ribosome. The 16S rRNA decodes the mRNA message by selectively binding acylated tRNAs [52], while the 23S rRNA catalyzes peptide bond formation [53] (Fig 4A, PDBs 2WDK and 2WDL [54]). Not surprisingly, the ribosome is an important drug target, with over 50 bacterial antibiotics developed to date [10].

**Fig 5:**
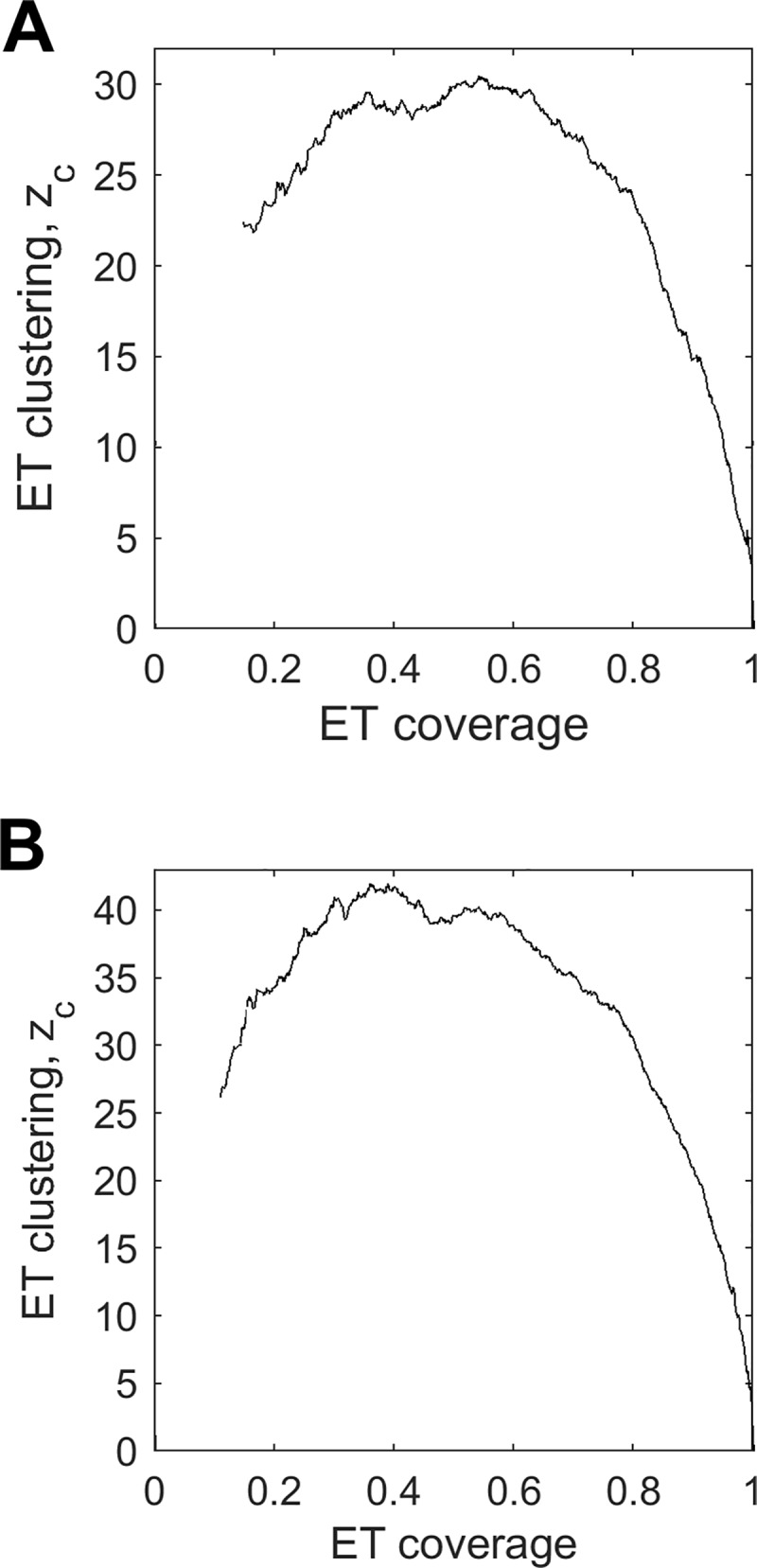
ET nucleotides cluster in the ribosome. Clustering z-score *z*_c_ for 16S in (A) and 23S rRNA in (B). The high clustering z-scores are indicative of large functional cores.

**Fig 6:**
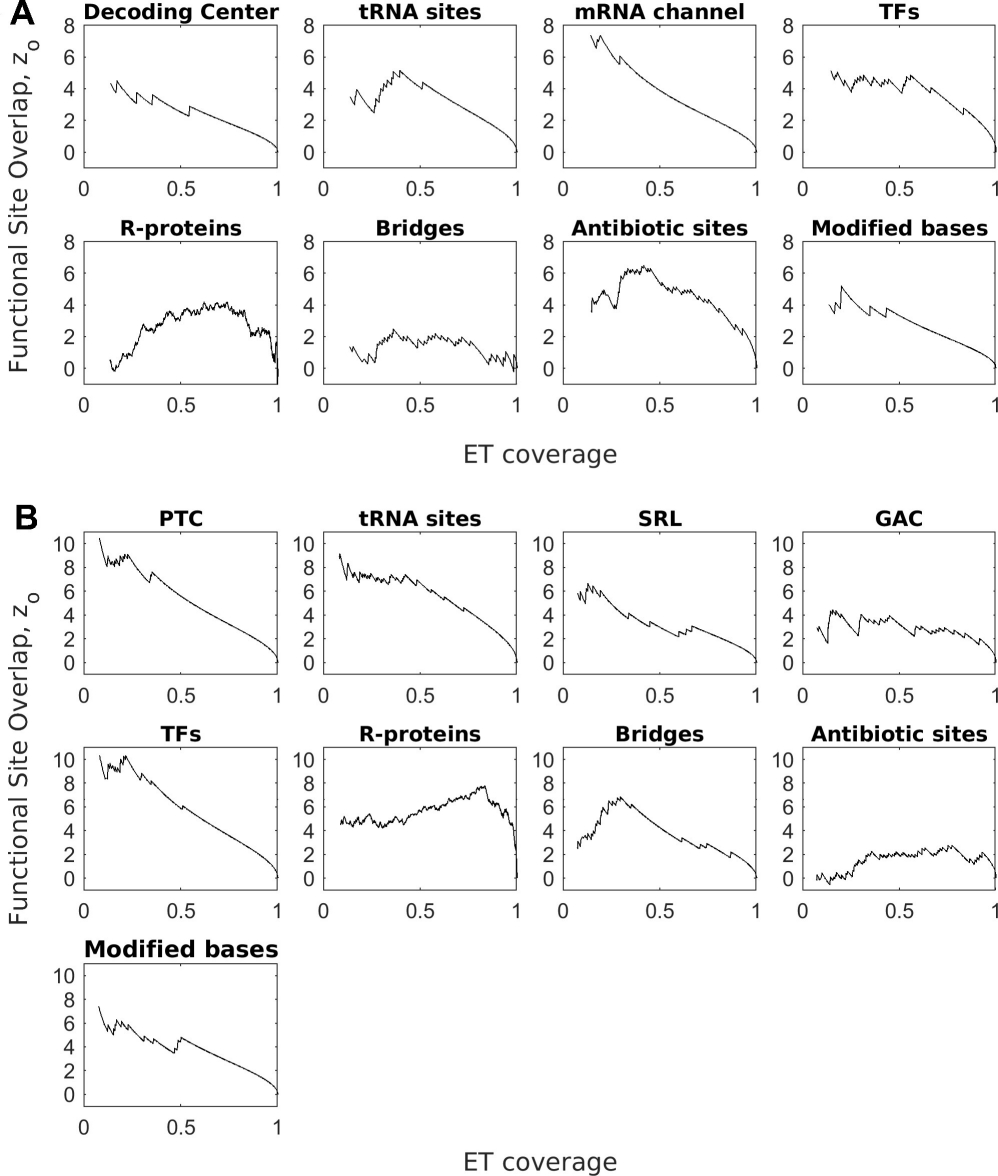
ET predicts functional sites in the ribosome. (A) Overlap between ET bases and functional sites in 16S RNA. The sites are—decoding center (DC), tRNA binding sites, mRNA channel, translation factor (TF) binding sites, structural protein contact sites, antibiotic binding sites, and modified bases. (B) Overlap between ET bases and functional sites in the 23S rRNA. The sites are–peptidyl-transferase center (PTC), tRNA binding sites, sarcin-ricin loop (SRL), GTPase-associated center (GAC), translation factor binding sites, structural protein contact sites, antibiotic binding sites, and modified bases. Corresponding ROC AUC are shown in S5 Fig. The nucleotides defining each functional site are listed in S2 Table.

Traces for the 16S and 23S RNA were computed using curated alignments of bacterial rRNA provided as part of [55]. ET ranks, mapped onto the three-dimensional structure of the RNAs (Fig 4A), revealed that ET nucleotides clustered in the structure (mean ET clustering z-score $z_{c}^{35\%}=24.3$ in the 16S, and 32.8 in the 23S, Fig 5). Furthermore, ET nucleotides broadly overlapped major functional sites in the ribosome–the peptidyl-transferase center in 23S, and the decoding center in 16S (relevant nucleotides marked in Fig 4B and 4C). Quantification of overlap for these and other major functional sites is summarized in Fig 6 (see S5 Fig for corresponding ROC curves, S2 Table for nucleotide definitions of each active site, and S4 Table for raw count of functional nucleotides recovered at ET threshold of 35%). In detail, in the 16S rRNA, ET bases overlapped the decoding center (mean $z_{o}^{35\%}=3.9$, AUC = 0.91), the tRNA E-, A-, and P- sites (mean $z_{o}^{35\%}=3.4$, AUC = 0.88), as well as the mRNA channel (mean ET overlap z-score $z_{o}^{35\%}=6.7$, AUC = 0.98). In the 23S rRNA, the ET cluster overlapped the peptidyl-transferase center (mean $z_{o}^{35\%}=8.5$, AUC = 0.94), the tRNA binding sites (mean $z_{o}^{35\%}=7.4$, and AUC = 0.86), as well as the GTPase-associated center (mean $z_{o}^{35\%}=2.9$, AUC = 0.72) and the sarcin-ricin loop (mean $z_{o}^{35\%}=5.1$, AUC = 0.85). These data show that ET detects the critically-important active sites in the ribosome.

Next, we tested whether ET bases also overlap protein binding sites (refer to panels in Fig 6A and 6B). First, we examined the binding sites of bacterial translation factors (including IF1, EF-Tu, EF-G, RF1, and RF3 for which high-resolution structures are available). They transiently bind the ribosome and are required for timely initiation, elongation and termination of translation. We defined binding sites as all rRNA bases within 4Å of the protein, and found that ET bases overlap TF binding sites with mean ET z-score $z_{o}^{35\%}=4.8$ in the 16S rRNA and 9.2 in the 23S (AUC = 0.84 and 0.94 respectively). Next, we tested the binding sites of structural ribosomal proteins, which serve as scaffolding and are enfolded by the rRNA during assembly [56]. ET bases overlap these contact sites with mean ET z-score $z_{o}^{35\%}$ = 0.96 and 4.6 in 16S and 23S respectively (AUC = 0.62 and 0.63). As expected, overlap between ET bases and translation factor binding sites is markedly higher than the ET overlap with structural protein contact sites. This is in line with the expectation that structural proteins are not as critical to function. Unexpected is the discrepancy between ET overlap for r-protein sites in 16S and 23S rRNA ($z_{o}^{35\%}$ of 0.96 vs 4.6). Interestingly, r-proteins in the 16S occupy 42% of all nucleotides, compared to only 29% in the 23S rRNA. This implies higher specificity of binding in the 23S subunit, resulting in better overlap with ET nucleotides. These data reflect ET’s sensitivity to the evolutionary pressure exerted across the ribosome. ET clearly separates more important sites, such as the catalytic core and TF binding sites, from the r-protein sites. In summary, ET ranks of the nucleotides correlate strongly with their functional impact.

Next, we also tested the critical structural bridges [57] that connect the two subunits for overlap with ET nucleotides. We found that ET bases overlap the bridges in the 23S (mean $z_{o}^{35\%}=4.6$ and AUC = 0.82), but not the 16S subunit (mean $z_{o}^{35\%}=1.2$, AUC = 0.66). To explain this difference, we examined the molecular basis of the bridge contacts, and found that 22 out of 48 contacts on the 16S side are formed by the nucleotide phosphate backbone, compared to only 7 out of 30 on the 23S (the difference is significant with hypergeometric p-value of 0.03). Because phosphate contacts are non-specific (not depended on the identity of the base), the nucleotides in 16S bridges are not evolutionary constrained and are ranked lower by ET. These data show that while ET is able to detect critical structural elements, the underlying molecular determinants can produce exceptions to the ET model, similar to the earlier example of the catalytic mechanism in the hammerhead.

Because ET bases broadly overlap functional sites, we next asked: do known ribosomal antibiotics also target ET bases? To determine if ET recovers known antibiotic binding sites, we compiled a list of binding sites for 32 different antibiotics [11]. We quantified recovery of these sites by ET, and found that ET bases overlap antibiotic binding sites with mean ET z-score $z_{o}^{35\%}=0.25$ in 23S and 4.3 in the 16S (AUC = 0.62 and 0.80). While the data confirm that ET bases overlap strongly with antibiotic binding sites in the 16S rRNA, we asked why there was a lack of overlap in the 23S subunit. We examined the molecular basis for antibiotic action in 16S and 23S rRNA. We found that the 23S antibiotics mainly target the exit channel, which is a tunnel that traverses the subunit, and it is used to extrude the nascent polypeptide from the ribosome. Because the channel does not serve as a site for catalysis or binding, it is lined with nucleotides that are not under heavy selective pressure. As a result, 23S antibiotic binding sites have a modest overlap with ET bases. In contrast, we found that in the 16S RNA, antibiotic families primarily target the mRNA binding channel and the decoding center, which, as we already showed, are primarily composed of high-ranked ET bases. Thus, the molecular mechanism of antibiotic action is in line with ET nucleotide ranking.

Next, we tested ET's ability to detect bases that do not necessarily belong to an established active site or binding interface, but are nevertheless hypothesized to be important: modified bases and those with known deleterious mutations We first examined modified bases; nascent rRNAs can be post-transcriptionally modified, and at least 34 nucleotides in the ribosome carry modifications [58]. While the exact role of modified bases is unclear, ribosomes assembled from unmodified rRNA are less active than wild type [59]. ET overlaps modified bases with mean ET z-score $z_{o}^{35\%}=4.0$ in 16S and 5.7 in the 23S (AUC = 0.93 and 0.87), suggesting that these bases evolved to perform a function in the large subunit. In addition to the modified bases, we examined nucleotides with known deleterious mutations. We compiled a list of mutations available on the Comparative RNA Website database [60], and sorted them into unambiguously deleterious or benign. Applying ET, we see that while both cohorts overlap with ET bases, there is clear separation between the two categories of mutations (S6 Fig). In the 16S, ET bases overlap with deleterious mutations more frequently than with benign mutations (mean z-score $z_{o}^{35\%}$ = 4.5 and AUC = 0.92 for deleterious mutations, and $z_{o}^{35\%}=1.9$ and AUC = 0.69 for benign). In the 23S subunit, the difference is also present (mean z-score $z_{o}^{35\%}=6.1$ and AUC = 0.94 for deleterious mutations, and $z_{o}^{35\%}=2.9$ and AUC = 0.81 for benign). Nucleotides with benign mutations rank consistently lower than nucleotides with lethal mutations. These data further point at a clear connection between evolutionary importance, as measured by ET, and functional impact.

**Fig 7:**
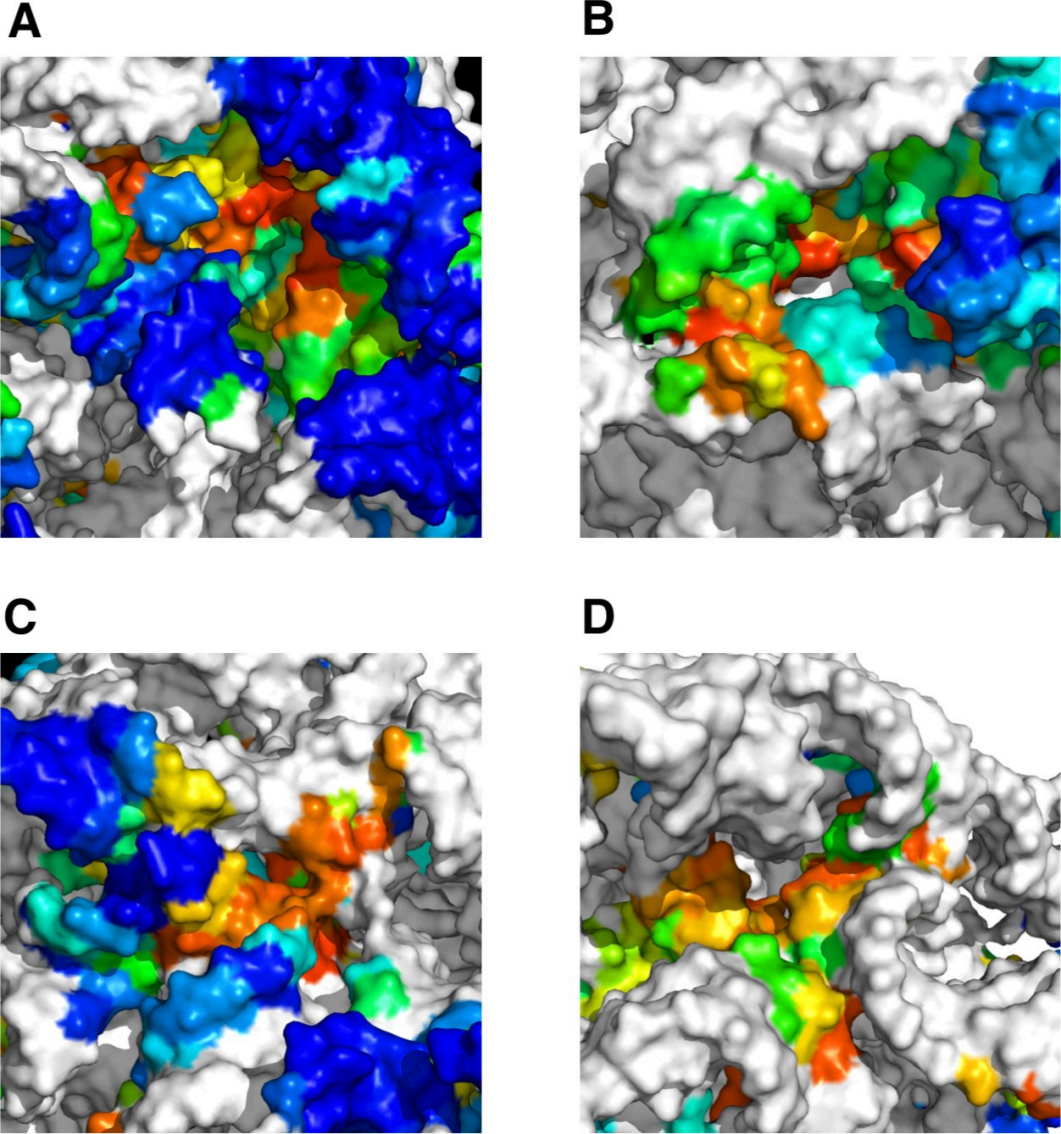
ET clusters of unknown functional significance. Clusters (A-C) in the 23S rRNA could serve as binding sites for regulatory proteins, while cluster (D) in the 16S is close to a translation factor binding site, and therefore could play a role in translation. Nucleotides in white are either known functional sites, or within 10Å of a r-protein, and therefore excluded from analysis.

Finally, we examined ET clusters that do not overlap with a known functional site. From the ribosomal structure, we excluded all nucleotides composing known sites, as well as all nucleotides within 10Å of a r-protein. This exclusion analysis produced 4 clusters of high-ranked ET nucleotides on the surface of the ribosome, three in the 23S and one in the 16S (Fig 7). The ET nucleotides in these clusters (listed for each cluster in S5 Table) are undocumented in the literature and carry no obvious functional significance. We propose that clusters in the 23S could serve as sites for binding of regulatory proteins and chaperones, or as sites of ribosomal processing during maturation and assembly. Meanwhile, the ET cluster in 16S is located adjacent to helix h5, which acts as a binding site for several translation factors [61, 62]. It is therefore possible that nucleotides in this cluster are involved in allosteric regulation of translation. These data show that ET-guided structural analysis can suggest sites of interest even in the well-studied systems such as the ribosome.

In summary, we discovered that ET nucleotides cluster on the structure of the ribosome, and that the core ET cluster clearly defines the major functional sites of the molecule. Additionally, ET ranking also suggests protein and antibiotic binding sites. We also show that higher-ranked ET nucleotides are enriched for inactivating mutations and post-transcriptionally modified bases. In detecting these sites, ET is more accurate than conservation (S7 Fig). Finally, because these data indicate that the ET model applies to the ribosome, we suggest several potentially novel sites.

### Generalizing the model

The hammerhead and ribosome case studies are consistent with two fundamental ET properties: that top-ranked bases cluster structurally, thereby revealing functional sites. To assess the generality of these features, ET was next tested on RNA families in the Rfam database. We selected 1070 RNA families that had at least 10 canonical sequences in their Rfam seed alignment. This set of RNAs included a broad selection of classes, including riboswitches, tRNAs, RNAzymes, viral particles, small regulatory RNA, and lncRNA (Fig 2A). Additionally, among these are 71 families that can be paired with at least one high resolution structure. Each alignment was traced, and the trace was evaluated for clustering among ET bases as a function of ET coverage.

**Fig 8:**
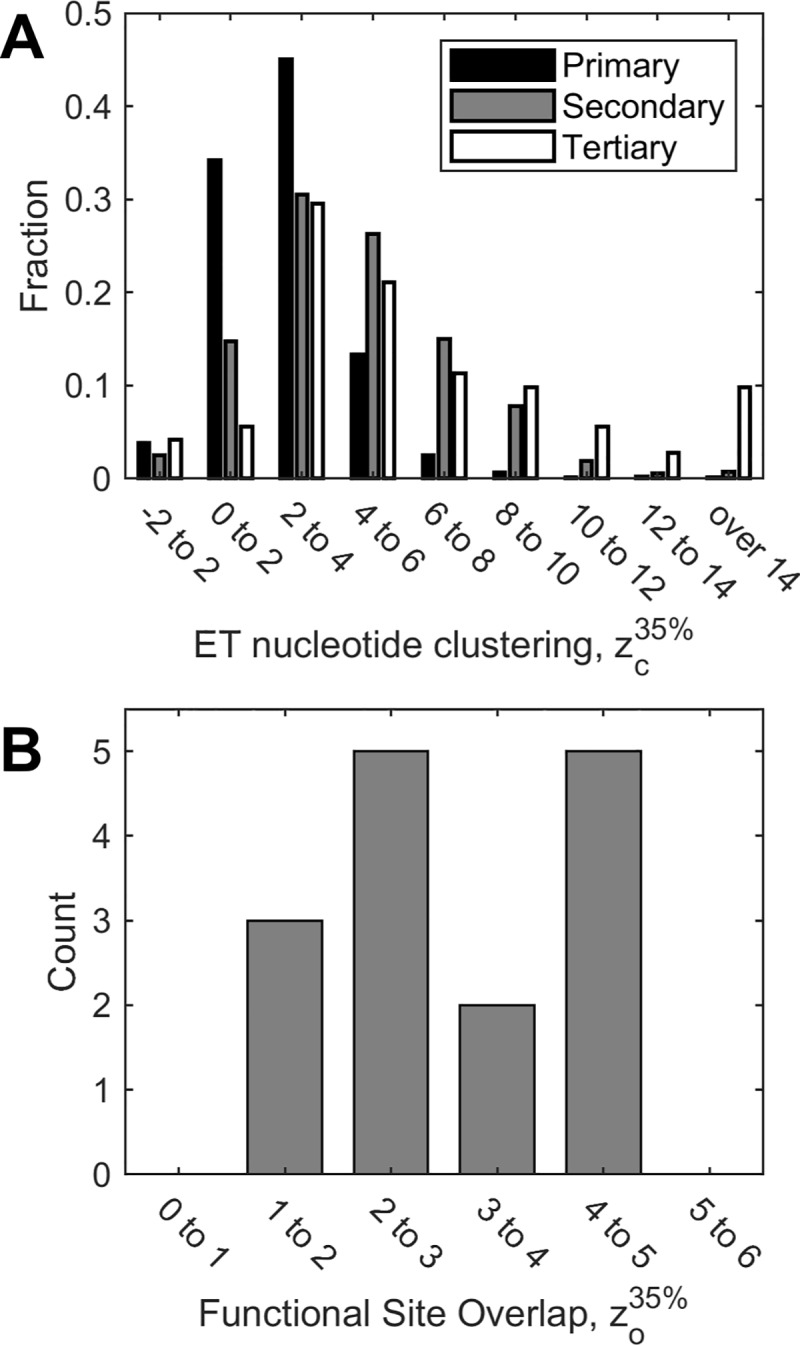
Clustering of ET nucleotides, and their overlap with functional sites is general. Z-scores above 2 indicate statistically significant clustering and overlap. Shown in (A) are aggregate ET clustering data for 1070 RNA molecules. ET clustering is detected in 62% of primary sequences, 83% of secondary structures, and 91% of tertiary structures. In a subset of 15 of these molecules (B), we measure overlap with known functional sites. In 12 of 15 test cases, overlap is significant.

We first evaluated the high-resolution structured set, and found that on average ET nucleotides recovered 24% of all tertiary structure contacts, compared to 13% that would be recovered by a random selection (see S6A Table). In 64 out of 71 RNAs, this corresponded to a mean clustering z-score $z_{c}^{35\%}$ greater than 2, indicating that ET is detecting clusters of evolutionarily important nucleotides (Fig 8A, white). This set consisted of well-ordered full length structures including riboswitches, RNAse P, catalytic introns, ribozymes, and rRNAs (Fig 2B**)**. Notably, ribosomal and splicesomal RNA displayed larger *z*-scores compared to the rest of the set (14 or more), suggesting large and evolutionary-important core functions. We then examined the 7 Rfam families that did not show structural clustering by ET nucleotides. Three of those families were small viral structures (approximately 30 nucleotides in length) found in the Human Immunodeficiency Virus (HIV) RNA. Their sequence alignments consisted of highly similar sequences (mean sequence identity 91%), and their narrow phylogenetic scope precluded meaningful ET analysis. By contrast, the average mean sequence identity in successful examples was 64%. Finally, in each of the remaining 4 families that performed poorly, clustering could not be fully assessed, because their best matched structures were a fragment of the whole length molecule. Overall, however, these data show that the model is in keeping with observations in 90%of the fncRNAs we were able to test. Failures rarely but consistently associated with missing structural context, or a deficit of evolutionary information due to a lack of sufficiently divergent sequences.

Next, in order to test ET for RNAs without known three-dimensional structures, which is 93% of our test set, ET base clustering was assessed in the primary sequence (1-dimension), and in the secondary structure models provided by Rfam (2-dimensions). 83% of secondary structures (Fig 8A, gray) displayed ET clustering with $z_{c}^{35\%}$ above 2 (in line with our observation in the tertiary set, ET nucleotides recovered an average of 27% of the secondary structure contacts, with 17% expected by random chance, see S6B Table). The few outliers with large z-scores, once again, were rRNA and spliceosome subunits. ET clustering could also be detected in the majority of primary sequences, with 62% reaching $z_{c}^{35\%}$ of 2 (Fig 8A, black). These data show that while secondary and primary structures lack the nuanced three-dimensional context, they nonetheless reveal clusters of ET nucleotides. One possible application of this property is to use ET clustering in predicted secondary structures to distinguish between poor and robust models.

Finally, of the 71 RNA families tested for three-dimensional ET clustering, we selected 15 for functional site analysis. These included the hammerhead ribozyme, the two ribosomal subunits, tRNA, RNAse P, group I self-splicing introns, and 9 riboswitches. For each of these molecules, we searched the literature for the canonical functional sites (see S1 Table), and then computed their overlap with ET bases (Fig 8B). In 12 of 15 cases, functional site overlap z-score $z_{o}^{35\%}$ was above 2.0. In two cases, the THF riboswitch and the PreQ1 riboswitch (RF01831 and RF00522), overlap approached significance with $z_{o}^{35\%}$ = 1.86 and 1.84. Finally, functional site overlap z-score $z_{o}^{35\%}$ was 1.44 for the FMN riboswitch (RF00522). Interestingly, its seed alignment contained a number of misaligned sequences; removing them, and retracing, raised $z_{o}^{35\%}$ from 1.44 to 1.9.

Together, these analyses of ET clustering and overlap suggest that the ET model is general and applicable to a wide range of functional RNAs.

### Optimizing sequence selection improves performance

Since, in RNA, ET fulfills the same clustering and functional site overlap properties as in proteins, perhaps that likewise improving the quality of the structural clusters can guide improvements to the quality of functional site predictions? In proteins the two correlate strongly. As a result, improvements in ET clustering can be used to optimize sequence selection, which in turn produces better functional site recovery [41], with important practical ramification in optimization of analyses [42].

To test this hypothesis, we assessed two different metrics of cluster quality: ET clustering z-score, as described earlier, and the ET smoothness. ET smoothness is the cumulative ET rank difference between all neighboring nucleotides in the structure (meaning lower absolute values for smoothness corresponds to a smoother distribution of ranks). This measure reflects smoothness of evolution over the entire structure, and is a more holistic metric than the mean ET base clustering [43].

**Fig 9:**
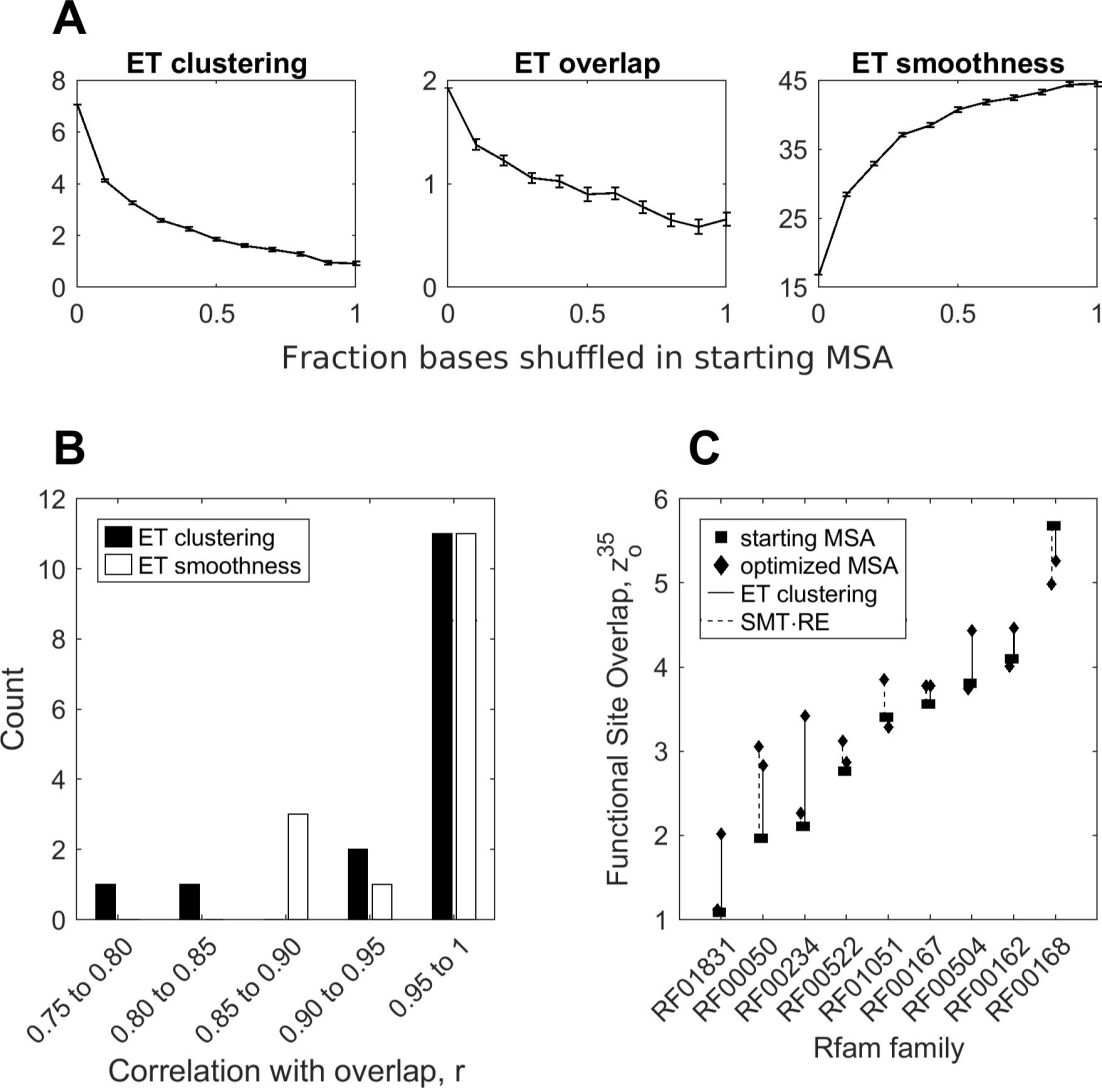
Optimization of input alignments via ET clustering and smoothness improve overlap. Degrading the glmS alignment in (A) shows that clustering and smoothness are correlated with overlap. Applying this analysis to the 15 molecules with annotated functional sites shows that the correlations are general (B). This suggests that sequence selection can be optimized to produce a more effective ranking of ET. This is shown in (C), where we use two different clustering measures, to select alignments that produce better functional site predictions than starting alignments.

We tested the relationship between the clustering metrics and the quality of prediction in 15 RNA families with curated functional sites. For each family, we generated a set of 1,000 alignments by randomly shuffling bases in the original alignment. We traced the alignments, and measured their smoothness, and their mean overall clustering and overlap z-scores, 〈*z*_*o*_〉 and 〈*z*_*c*_〉. We then binned the alignments by their shuffle rate, and averaged the scores, as shown in Fig 9A for glmS riboswitch. As seen in the glmS example, as we introduce errors into the alignment, ET overlap, clustering, and smoothness deteriorate in highly correlated manner. Across the 15 test cases, mean correlation between ET overlap and clustering was r = 0.95, and r = -0.96 between overlap and smoothness (Fig 9B).

The correlation between structural quality of the trace and overlap has practical implications, because smoothness and ET clustering can be used as indirect measures of trace fidelity. By optimizing trace alignments to maximize smoothness (or ET clustering), we, presumably, also maximize accuracy of active site prediction. We tested this hypothesis in the nine test riboswitches. For each riboswitch, we generated a starting alignment, distinct from the seed, and based on 500 sequences chosen at random from the family’s full sequence repository on Rfam. Using these alignments as reference, we then generated for each riboswitch an ensemble of 5,000 alignments by removing a random number of sequences *0< n< N–20* (where *N* is the size of the alignment) from the reference. We then traced each alignment, and computed its ET smoothness, ET clustering z-score $z_{c}^{35\%}$, and ET overlap z-score $z_{o}^{35\%}$. Finally, we chose from the random ensemble, the alignment that has the smoothest ET, or the highest ET clustering. We then compared overlap z-scores produced by the smoothness- and clustering-optimized alignments to the original Rfam alignments.

Startlingly, we found that in 8 of 9 cases, optimization via ET smoothness led to decrease in performance, with a mean reduction of 13%. To explain this behavior, we examined the smoothest alignments, and found that the optimization selected alignments consisting of a small number of highly invariant sequences. These alignments sacrificed phylogenetic diversity as a trade-off for a very smooth, yet uninformative, ET ranking of highly conserved nucleotides. To address this problem of narrow phylogenetic scope, we introduced Rank Entropy (RE), $\sum_{r}^{N}\;fr\;log(fr)$, which measures the frequency (*fr*) of each ET rank (*r*) in the alignment. RE is maximum when each column is assigned a unique rank, and is zero when the alignment is entirely conserved.

Normalizing RE and ET Smoothness to 1, and then using their product as a single measure (RE×SMT), rescued optimization performance in 8 of 9 cases, yielding mean improvement of 9% in prediction quality (Fig 9C, dashed lines). Finally, we tested mean ET clustering as an optimization metric. We found that maximizing mean ET clustering z-score produced alignments that yielded an average improvement of 24% (Fig 9C, solid line). ET clustering z-score performed better than smoothness-based metrics, because it expresses significance of ET nucleotide clustering relative to the remaining structure. When the conserved ET bin dominates the trace (ET nucleotides are more likely to cluster), this results in a greater expected clustering weight, a larger standard deviation, and as a result a lower clustering z-score, making this measure more sensitive than raw ET smoothness.

Notably, the optimized full alignments also outperformed the manually curated Rfam seed alignments, which are used in the baseline functional site prediction experiment in Fig 8. While the full alignments we used for optimization are already better than seed (10% improvement in z-score of overlap, due to a more diverse sequence set), optimization further increases the gap in performance. Optimization via rank-adjusted smoothness produced alignments that are better than seed at detecting the functional site in 6 of 9 cases (mean improvement of 21%), and optimization via ET clustering produced better alignments than seed in all 9 cases (30% mean improvement over seed). To further test if we can optimize manually curated alignments, we applied the two optimization techniques to the multiple sequence alignments used in the ribosomal case study. Measuring accuracy over the aggregate of all functional sites, we found that optimization by rank-adjusted smoothness improved prediction accuracy in the small subunit from mean 0-35% ET overlap z-score z = 2.38 to z = 2.60, and in the large subunit led to a decrease from z = 6.95 to 6.80. However, optimization via ET clustering elevated accuracy in both subunits, to z = 3.32 in the small subunit, and z = 7.20 in the large subunit accordingly. Together, these data show that optimization of input alignments is both possible and useful.

In summary, sequence selection can be optimized in order to achieve better active site recovery. Input sequences are the principal factor that affects the quality of the trace. Using ET clustering and RE-smoothness as indirect measures, we can remove sequences that are either too phylogenetically distant or erroneous. In this manner, we can elevate trace quality and more accurately predict the active sites.

### Conclusion

Over 50 years ago, Carl Woese and Francis Crick hypothesized that RNA could serve as a precursor to DNA and proteins [63], and since then, RNAs have been found to perform a variety of roles within the cell. Here, we show that the similarity between functional RNAs and proteins is not just a subject of anecdotal likeness. We argue that this similarity is based upon the fundamental principle of selection that governs evolution of function in both RNAs and proteins. In the same manner as evolutionarily important amino acids in proteins, evolutionarily important RNA nucleotides evolve in compact, non-random clusters that inform on the function of the molecule. As we have shown in a number of examples, including the hammerhead and the ribosome, these clusters correspond to catalytic sites, ligand-binding pockets, and molecular interfaces, and are enriched for inactivating mutations and modified nucleotides. These basic properties underline the relationship between sequence, structure, and function in structured RNAs, and suggest that it is possible to identify novel functional sites in structured RNA molecules using Evolutionary Trace analysis.

Furthermore, we demonstrate that there is a quantifiable correlation between cluster formation and recovery of functional sites. This correlation is directly informed by ET clustering and ET smoothness, which measures the evolutionary history similarity in nearby amino acids or nucleotides. In structured RNA, just like in proteins, evolution tends to minimize rank differences between neighboring nucleotides. This leads to formation of smooth clusters that inform function. In practice, this property allows us to maximize prediction accuracy of ET by constructing alignments that maximize spatial clustering and smoothness of ET nucleotides.

Interestingly, we also find that that there is some complementarity between ET analysis and covariation. To show this, we compared ET to R-scape [64], a covariation analysis tool, in bacterial RNAse P. We applied R-scape to the alignment of RNAse P sequences, and found 74 pairs of bases (148 nucleotides) with significant covariation (R-scape e-value < 0.05). We directly plotted R-scape covariation weights of these 148 nucleotides against their ET weights, shown in S8A Fig. We immediately find a correlation between ET score and R-scape score (correlation coefficient r = 0.47), indicating that highly covariant positions will tend to have a low ET score, and vice-versa. Secondly, as shown in S8B Fig, we find very little overlap between the top ET nucleotides and the 148 high covariation nucleotides. Finally, these two mostly non-overlapping nucleotide groups appear to modulate different features, as shown in S8C Fig. These data show that ET scores and R-scape covariation scores are highly complementary in this example, and we leave for a future study a deeper consideration of these comparisons to understand how well they generalize.

As this study has focused on well-known structured RNAs, it is not entirely clear whether ET properties are common to all RNA classes, especially novel classes such as lncRNA which have a tenuous link between sequence, structure, and function. However, in cases where the RNA of interest is well-represented by a set of diverse homologs, researchers will be able to use ET as a guide to target nucleotides for functional analysis. We expect the need for such analyses to grow in the future, as the scope of RNA research continues to expand. Lastly, some properties elucidated here for RNA could be translated directly to other polymers, particularly DNA. The ET scoring schema for scoring evolving polymers is generalist, and can be used on genomic sequences, to locate functional loci in the non-coding regions.

## Supporting information

S1 Fig Tree traversal and sequence group selection in ET. First, we number nodes from 1 to N-1 (where N is the number of sequences), according to their position relative to the root. Then, we iterate through each node in ascending order, and separate the tree into groups according to tree bifurcation. Because the tree is binary, the number of groups corresponds to current node position. Accordingly, when ET is evaluating the root (n = 1), all sequences belong to a single group, as shown in (A). As we move to node n = 2, there are 2 sequence groups (B), node n = 3 corresponds to 3 sequences groups (C), and so on, until we arrive at the last node, and the number of sequence groups is N-1 as shown in (D)
(TIFF)

S2 Fig ET identifies functional sites in the hammerhead (ROC AUC). ROC AUC measure of prediction accuracy for all sites combined (A), the core site (B), and the distal loops (C), is in agreement with ET overlap z-scores.
(TIFF)

S3 Fig ET outperforms conservation in detecting distal loops of the hammerhead. Overlap z-score shows that both ET and entropy identify the conserved catalytic core of the molecule (A). However, only ET identifies the distal region, which lacks obvious conservation (B). Represented as ROC curves in (C) and (D), the data support the same conclusion.
(TIF)

S4 Fig Nucleotide conservation in the hammerhead. Conservation scores are assigned according to Shannon Information Entropy, normalized to 0 to 100% coverage scale. Note that compared to ET mapping in Fig 3A, the distal regions are not as highly ranked.
(TIFF)

S5 Fig ET detects functional sites in the ribosome (ROC AUC). Broadly, measures of ROC AUC for the 16S rRNA (A) and the 23S rRNA (B) are in accordance with overlap z-scores.
(TIFF)

S6 Fig ET discriminates between deleterious and benign mutations in the ribosome. Both in (A) 23S and (B) 16S rRNA, nucleotides with benign mutations are scored lower by ET than nucleotides with lethal mutations, as shown by both overlap z-scores, left, and ROC AUCs, right.
(TIFF)

S7 Fig ET outperforms conservation in the ribosome. For each of the 17 ribosomal functional sites in our test set, we measured the difference in prediction accuracy between ET and conservation (Shannon information entropy). The four metrics of prediction accuracy used are (A) mean z-score of overlap for nucleotides bins ranked in top 0–35%, (B) z-score of overlap averaged over all rank bins (C) maximum overlap z-score, and (D) area under the ROC curve. While the scores agree, ET generally outperforms conservation.
(TIFF)

S8 Fig ET and R-scape are complementary in RNAse P. Direct comparison of scores in (A) shows that higher-ranked ET nucleotides tend to have low covariation, and vice-versa, with a correlation coefficient of r = 0.47 (note that the correlation coefficient is positive, instead of negative, because of ET percentile rank notation, where higher percentile rank corresponds to lower ET importance). In (B), we further show that there is very little overlap between the two nucleotide groups by plotting ET nucleotides (red) and the high covariation nucleotides (blue) on the structure of RNAse P. Note that only 16 nucleotides are found both in the ET group and the R-scape covariation group (shown in purple). Finally in (C), we include RNAse P substrate (tRNA in pink) and structural protein partner (yellow) to show that while ET nucleotides recovered functional sites, namely, the enzymatic site and the binding surfaces, the covarying nucleotides recovered the structural helices. Together these data show that in RNAse P, ET score and R-scape covariation score are complementary.
(TIFF)

S1 Table Rfam test set. Listed in S1A Table are the 1070 Rfam families traced in this study. In S1B Table is a subset of 70 families with high-resolution structures that make up the three-dimensional structure test set. Finally, in S1C Table is a subset of 15 Rfam families with annotated functional sites that we use in the functional site prediction test.
(XLSX)

S2 Table Annotation of functional sites in the ribosome. The table is an extension of S1C Table, and it enumerates nucleotides making up functional sites in the 16S and 23S rRNA.
(XLSX)

S3 Table ET recovers functional nucleotides in the hammerhead. This table details the hypergeometric analysis used to produce overlap z-scores in the hammerhead.
(XLSX)

S4 Table ET recovers functional nucleotides in the ribosome. This table details overlap z-score analysis for the ribosome at ET threshold of 35%.
(XLS)

S5 Table List of ribosomal nucleotides in the undocumented ET clusters. (XLSX)

S6 Table ET recovers structural contacts in Rfam families. This table details the clustering z-score analysis for the three-dimensional structure set (S6A) and the secondary structure set (S6B). In both cases ET nucleotides recover significantly more structural contacts than would be expected by random chance.
(XLSX)
