# Supplementary figures and images for: An Evolutionary Trace method defines functionally important bases and sites common to RNA families
Source: PLoS Comput Biol. 2020 Mar 24;16(3):e1007583. doi: 10.1371/journal.pcbi.1007583 (PMC7092961; doi:10.1371/journal.pcbi.1007583)

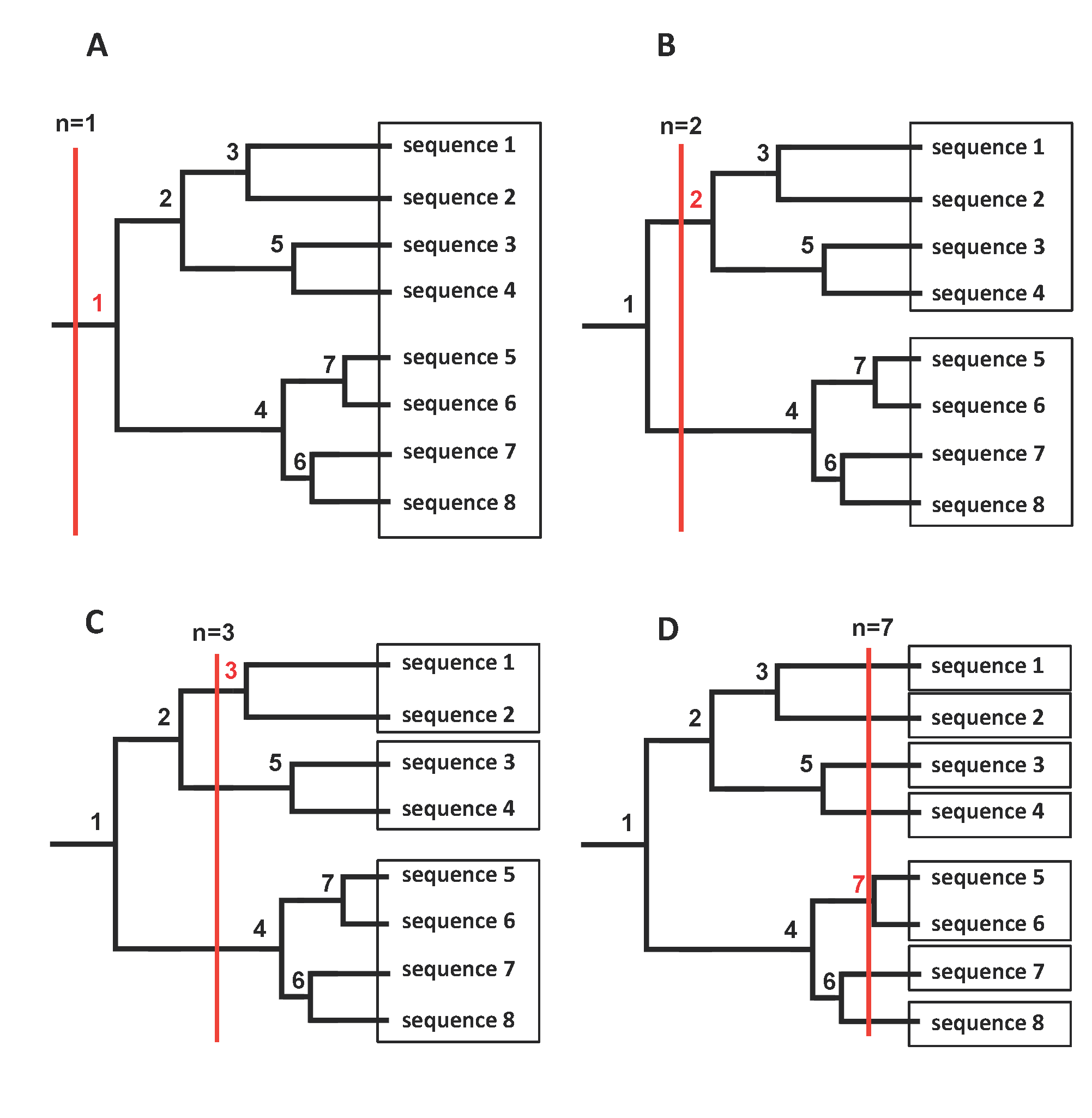

Supplement: S1 Fig — First, we number nodes from 1 to N-1 (where N is the number of sequences), according to their position relative to the root. Then, we iterate through each node in ascending order, and separate the tree into groups according to tree bifurcation. Because the tree is binary, the number of groups corresponds to current node position. Accordingly, when ET is evaluating the root (n = 1), all sequences belong to a single group, as shown in (A). As we move to node n = 2, there are 2 sequence groups (B), node n = 3 corresponds to 3 sequences groups (C), and so on, until we arrive at the last node, and the number of sequence groups is N-1 as shown in (D) (TIFF) [file pcbi.1007583.s001.tiff]

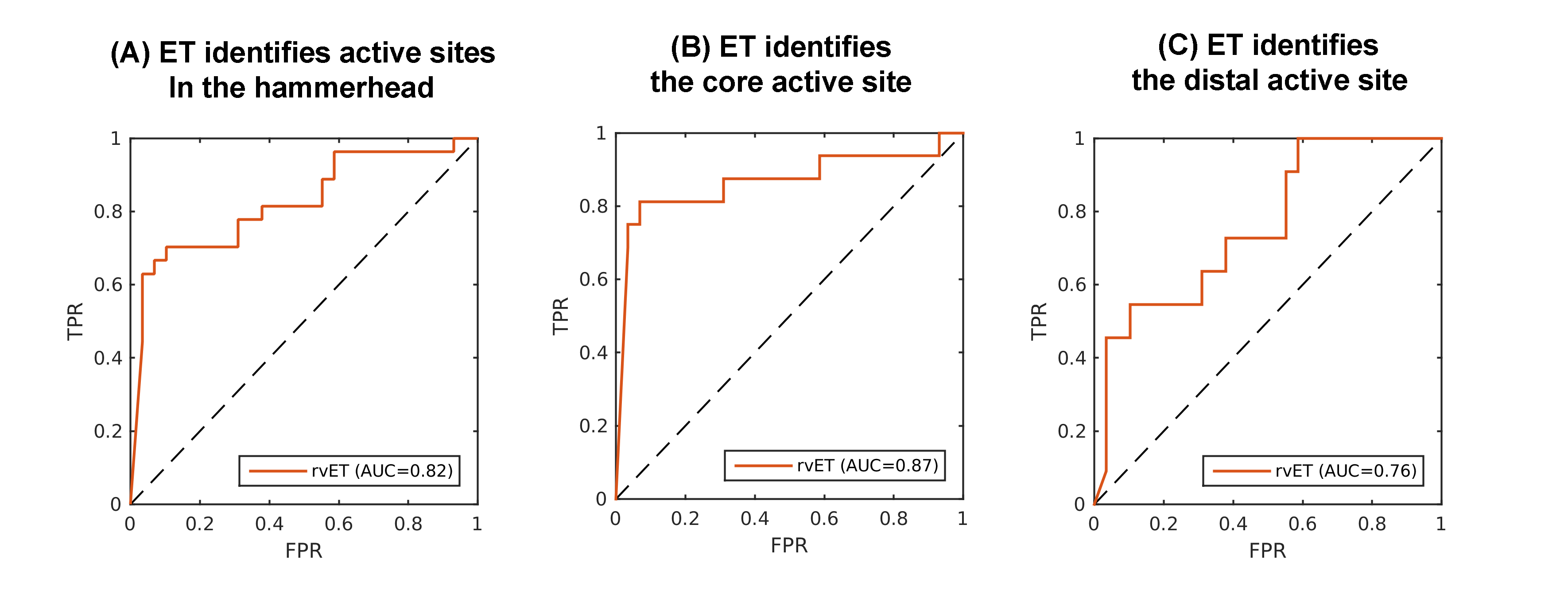

Supplement: S2 Fig — ROC AUC measure of prediction accuracy for all sites combined (A), the core site (B), and the distal loops (C), is in agreement with ET overlap z-scores. (TIFF) [file pcbi.1007583.s002.tiff]

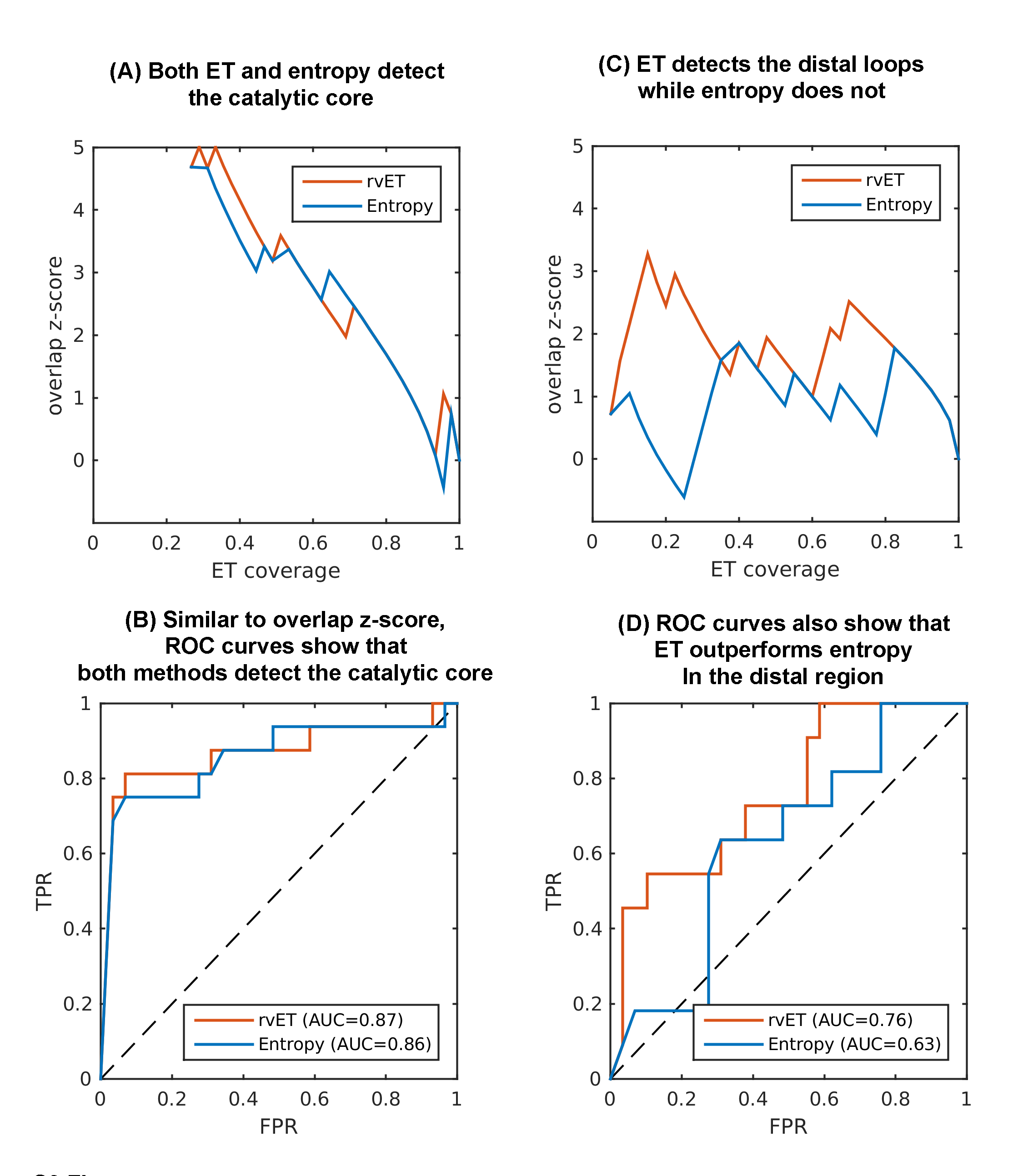

Supplement: S3 Fig — Overlap z-score shows that both ET and entropy identify the conserved catalytic core of the molecule (A). However, only ET identifies the distal region, which lacks obvious conservation (B). Represented as ROC curves in (C) and (D), the data support the same conclusion. (TIF) [file pcbi.1007583.s003.tif]

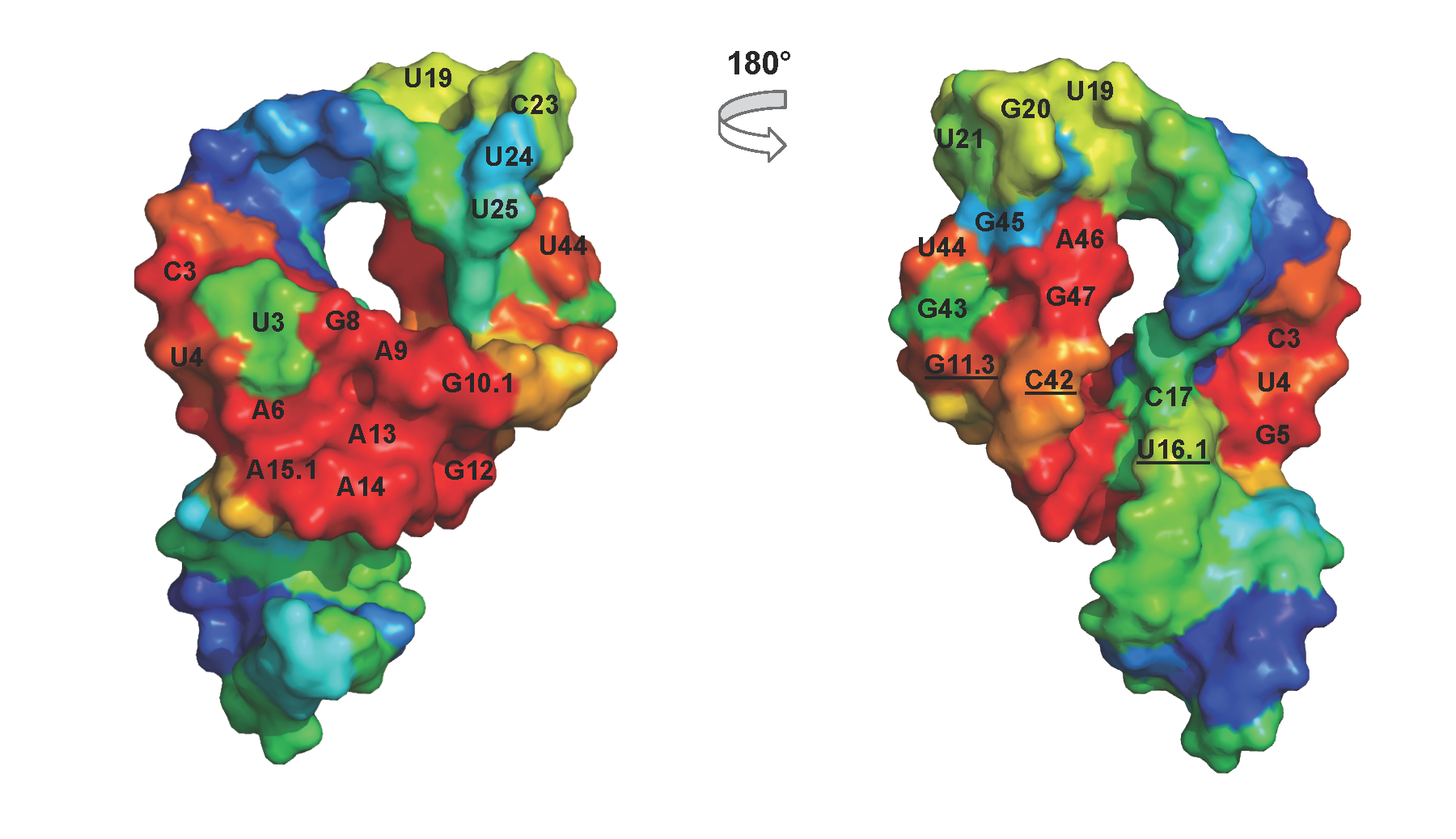

Supplement: S4 Fig — Conservation scores are assigned according to Shannon Information Entropy, normalized to 0 to 100% coverage scale. Note that compared to ET mapping in Fig 3A, the distal regions are not as highly ranked. (TIFF) [file pcbi.1007583.s004.tiff]

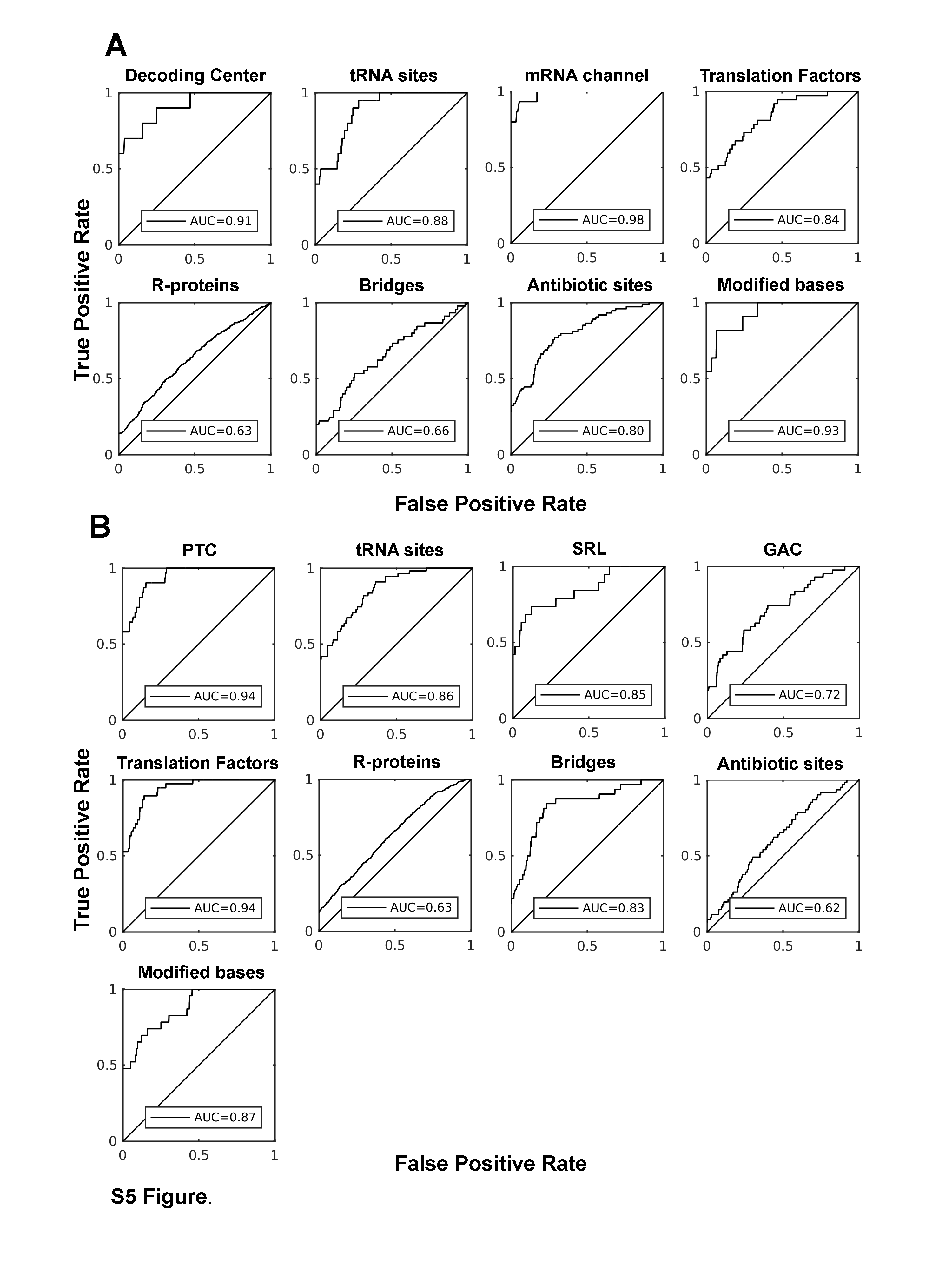

Supplement: S5 Fig — Broadly, measures of ROC AUC for the 16S rRNA (A) and the 23S rRNA (B) are in accordance with overlap z-scores. (TIFF) [file pcbi.1007583.s005.tiff]

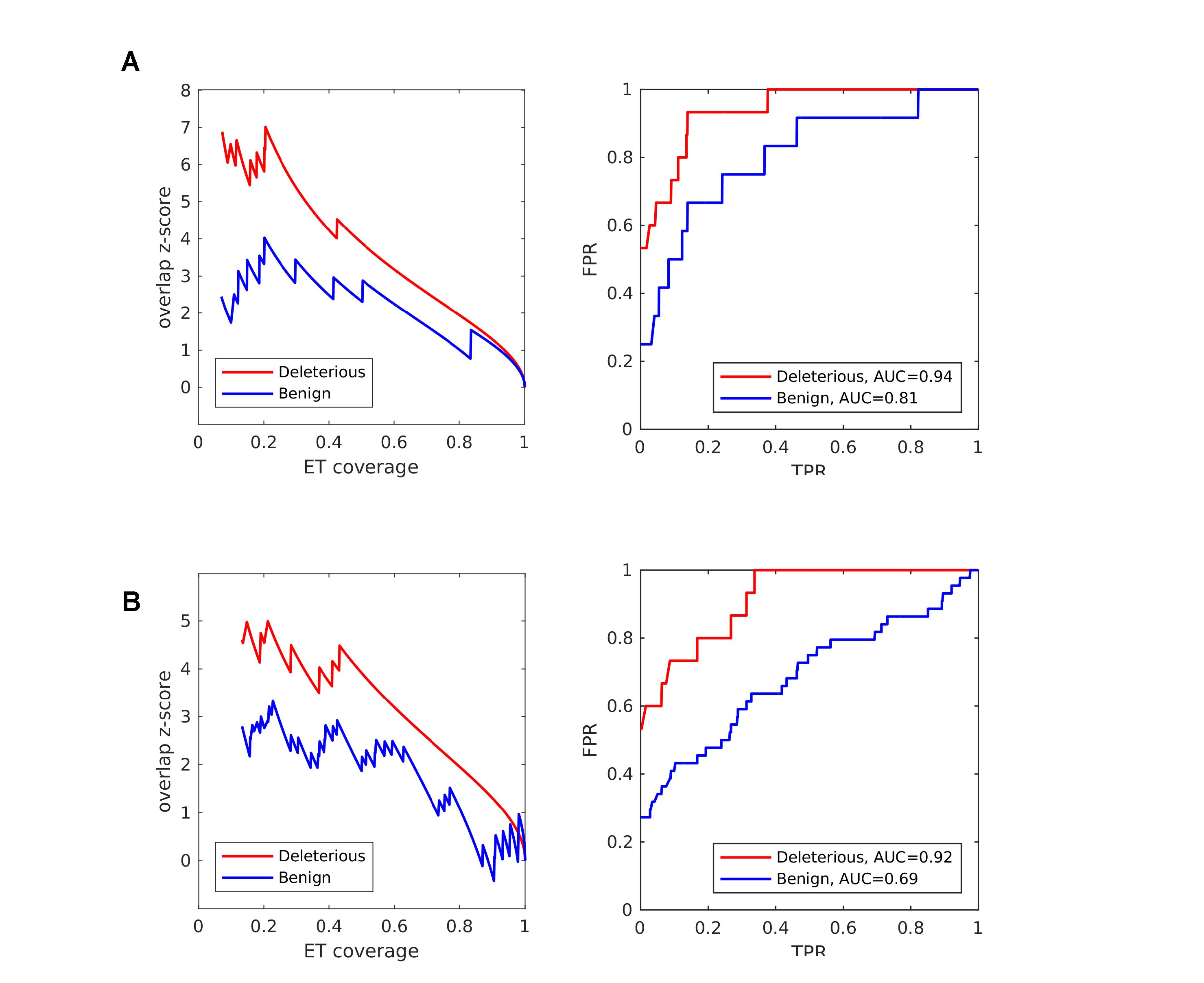

Supplement: S6 Fig — Both in (A) 23S and (B) 16S rRNA, nucleotides with benign mutations are scored lower by ET than nucleotides with lethal mutations, as shown by both overlap z-scores, left, and ROC AUCs, right. (TIFF) [file pcbi.1007583.s006.tiff]

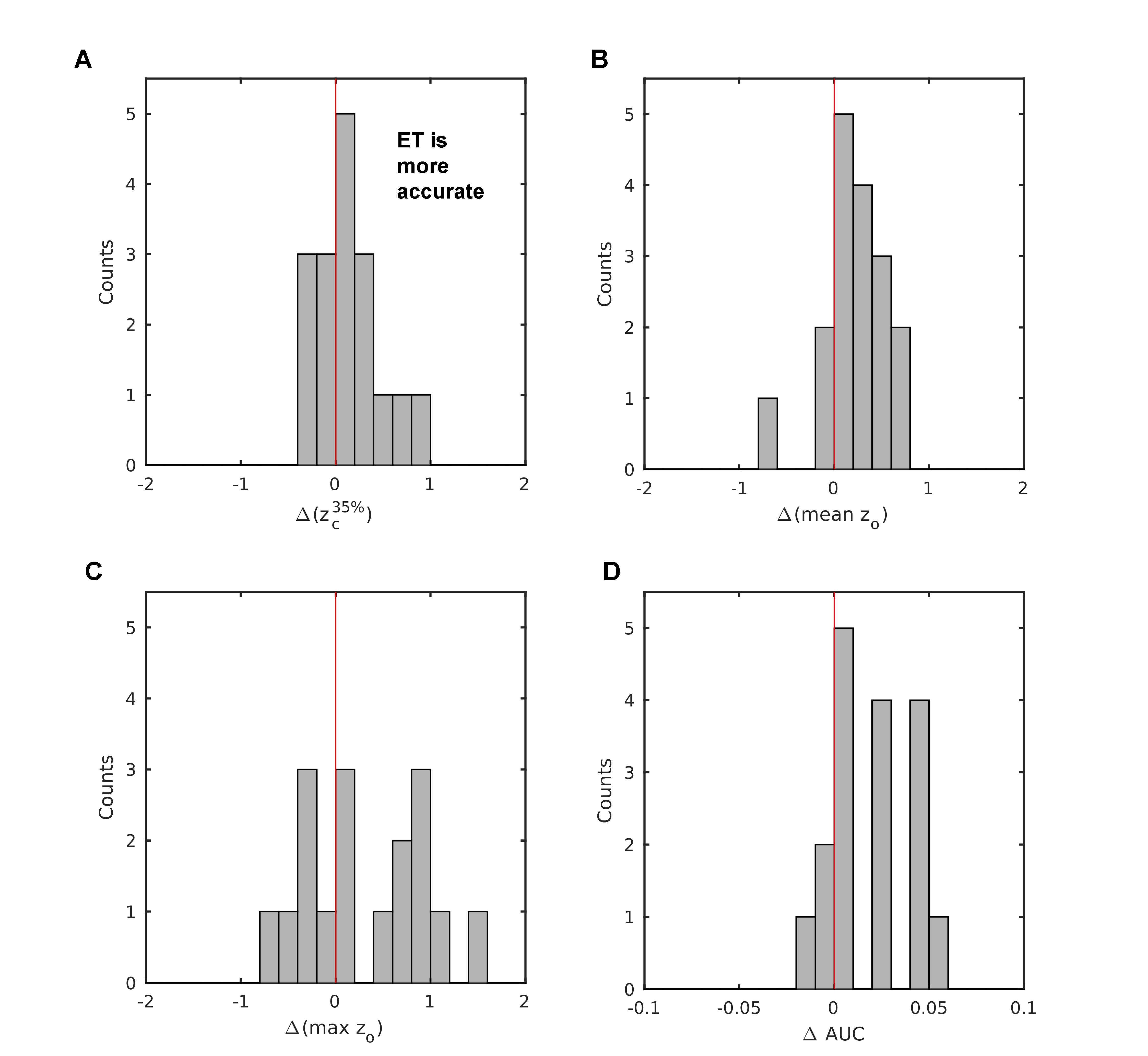

Supplement: S7 Fig — For each of the 17 ribosomal functional sites in our test set, we measured the difference in prediction accuracy between ET and conservation (Shannon information entropy). The four metrics of prediction accuracy used are (A) mean z-score of overlap for nucleotides bins ranked in top 0–35%, (B) z-score of overlap averaged over all rank bins (C) maximum overlap z-score, and (D) area under the ROC curve. While the scores agree, ET generally outperforms conservation. (TIFF) [file pcbi.1007583.s007.tiff]

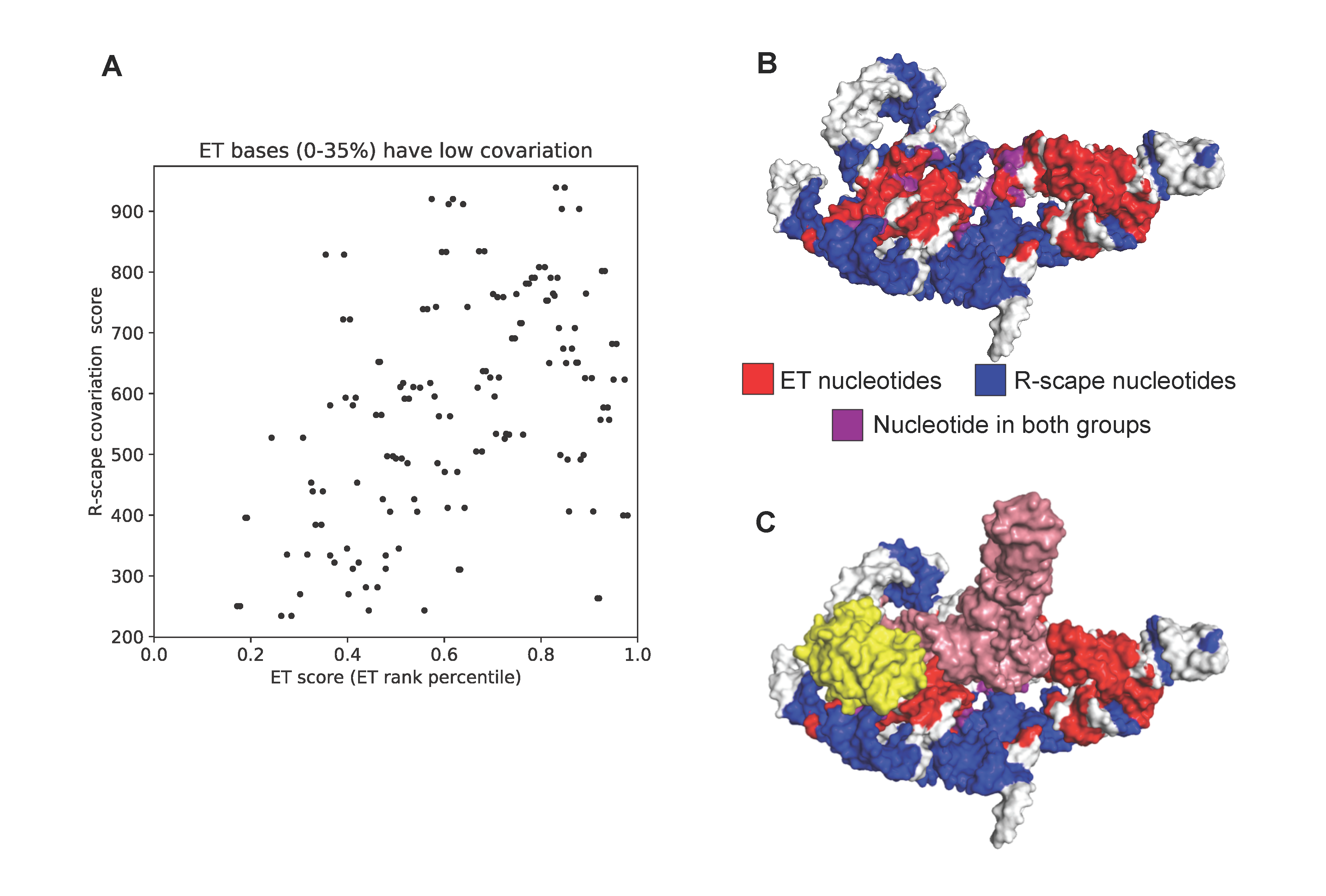

Supplement: S8 Fig — Direct comparison of scores in (A) shows that higher-ranked ET nucleotides tend to have low covariation, and vice-versa, with a correlation coefficient of r = 0.47 (note that the correlation coefficient is positive, instead of negative, because of ET percentile rank notation, where higher percentile rank corresponds to lower ET importance). In (B), we further show that there is very little overlap between the two nucleotide groups by plotting ET nucleotides (red) and the high covariation nucleotides (blue) on the structure of RNAse P. Note that only 16 nucleotides are found both in the ET group and the R-scape covariation group (shown in purple). Finally in (C), we include RNAse P substrate (tRNA in pink) and structural protein partner (yellow) to show that while ET nucleotides recovered functional sites, namely, the enzymatic site and the binding surfaces, the covarying nucleotides recovered the structural helices. Together these data show that in RNAse P, ET score and R-scape covariation score are complementary. (TIFF) [file pcbi.1007583.s008.tiff]
